# Machine Learning-Driven Discovery of Novel HER2 Inhibitors Through Integrated Virtual Screening and Molecular Dynamics Simulations

**DOI:** 10.3390/ph19081190

**Published:** 2026-07-29

**Authors:** Alhumaidi B. Alabbas, Safar M. Alqahtani

**Affiliations:** Department of Pharmaceutical Chemistry, College of Pharmacy, Prince Sattam Bin Abdulaziz University, Al Kharj 11942, Saudi Arabia; safar.alqahtani@psau.edu.sa

**Keywords:** breast cancer, machine learning, docking, MD simulation, RDF, Lipinski rule 5

## Abstract

**Background**: HER2 is a key oncogenic gene in breast cancer, involved in tumor progression, metastasis, and therapeutic resistance. This study aimed to find new HER2 inhibitors using a hybrid of machine learning (ML) and structure-based virtual screening (VS), combined with molecular dynamics (MD) simulations on various scaffolds. **Methods**: Four supervised molecular fingerprint classification models were trained on a dataset of 10,000 validated compounds from ChEMBL. Random Forest was the top model for screening a large compound library. Selected compounds underwent molecular docking in the HER2 ATP binding site, ADMET, drug likeness, toxicity analysis, and 200 ns MD simulations. Methods like PCA, FEL, hydrogen-bond analysis, DCCM, RDF, salt-bridge analysis, and MM/PBSA were used to assess binding stability. **Results**: Virtual screening identified three compounds, CHMEBL193865 (Lead-1), CHMEBL46740 (Lead-2), and CHMEBL151318 (Lead-3)—with better binding affinity and interaction profiles than the reference inhibitor. MD simulations showed stable protein–ligand complexes with RMSD values of 2.32–2.76 Å. Among these, Lead-2 was the most structurally stable, and Lead-1 had the most favorable binding free energy. All three compounds showed good drug likeness, ADMET properties, and low predicted toxicity. **Conclusions**: These findings support further in vitro and in vivo testing for developing new therapeutics against HER2-overexpressing breast cancer, highlighting two scaffolds with promising lead optimization potential.

## 1. Introduction

Breast cancer is a very heterogeneous and complicated malignant disease, which is the most common diagnosis in women all over the world [1]. The World Health Organization (WHO) states that the prevalence of breast cancer in terms of cancer-related mortality makes the further optimization of diagnostic and treatment methods extremely important. The interplay of the genetic, epigenetic, and environmental factors contributes to the pathogenesis of this malignancy and promotes the uncontrolled proliferation of cells, inhibition of apoptosis, angiogenesis, and distant metastasis [2]. About 15–20% of breast cancer patients are overexpressing HER2, a phenotype that is associated with a more aggressive phenotype, increased recurrence rates, and poorer clinical outcomes [3]. HER2- positive tumors are often resistant to traditional cytotoxic chemotherapy, and despite the introduction of antigenic therapies like trastuzumab, pertuzumab, and small-molecule tyrosine kinase inhibitors, intrinsic and acquired resistance remain risks to the long-term therapeutic efficacy [4]. Such a situation puts forward the need to identify new tactical approaches that can effectively target HER2 but avoid resistance mechanisms. As a result, HER2 has become a targeted drug in the treatment of breast cancer. Breast cancer remains the leading cancer diagnosis among women globally, seen in more countries than any other cancer type. In 2022, an estimated 2.3 million women were diagnosed with breast cancer, and about 670,000 women died from it—numbers that continue into 2025 as the primary global estimate as per the WHO report [2].

Existing treatment options for HER2-positive breast cancer include monoclonal antibodies, antibody–drug conjugates, and tyrosine kinase inhibitors, with each having a different mechanism of action [5]. Computational drug design has become a novel approach to the discovery and optimization of new HER2 inhibitors. Molecular docking and molecular dynamics (MD) simulations are structure-based methodologies that can be used to describe the nature of receptor–ligand interactions in silico, estimate binding affinities accurately, and define which residues are important when interacting with a ligand [6]. These methods enable scientists to promptly screen large collections of compounds, rank compounds to be empirically validated, and cycle molecular scaffolds to maximize efficacy and selectivity. Also, computational pharmacokinetic studies (ADME profiling) allow the early evaluation of drug-likeness properties, leading to a reduction in the risk of late-stage attrition. Since machine learning (ML) technologies complement the traditional computational strategies, they are transforming drug discovery by using large-scale datasets of biological, chemical, and clinical information [7]. Latent patterns may be revealed with the help of ML algorithms, bioactivity may be predicted, and molecular properties may be optimized to shorten the development of potent and selective HER2 inhibitors [8]. As an example, supervised learning models that have been trained on high-throughput screening data can be used to classify compounds as active or inactive, and regression models can be used to predict quantitative values (such as binding affinity). The combination of ML and computational chemistry not only makes the process of rational drug design more efficient but also makes experimental procedures less expensive and provides a platform of accuracy in oncology tailored to personal patient profiles [9]. The combination of computational modeling and ML provides a multiscale understanding of the biology of HER2. In addition to ligand–receptor connections, these techniques can also absorb omics data, such as genomics, transcriptomics, proteomics, and metabolomics, to determine biomarkers of predictive drug response or resistance [10]. Network biology frameworks also explain downstream signaling pathways, highlighting possible vulnerabilities that can be used therapeutically. This integrative approach not only allows for finding new drug targets but also optimizes combination therapy and individual treatment plans through thorough molecular phenotyping [11].

The objectives are (i) in silico screening of potential inhibitors; (ii) binding affinity assessments, docking, and MD simulations; (iii) prediction of pharmacokinetics; and (iv) using machine learning models to prioritize candidates based on their predicted efficacy and selectivity. To sum up, the merging of computational drug design and ML is an impressive paradigm in modern oncology studies. It is hoped that through an organized approach to integrating computational modeling, ML predictions, and biological intuition, this study can contribute to the creation of the next generation of HER2-targeted therapies, and ultimately, the objective is to positively impact patient outcomes in such a powerful form of breast cancer.

## 2. Results

### 2.1. Machine Learning (ML) Interpretation

**Figure 1:**
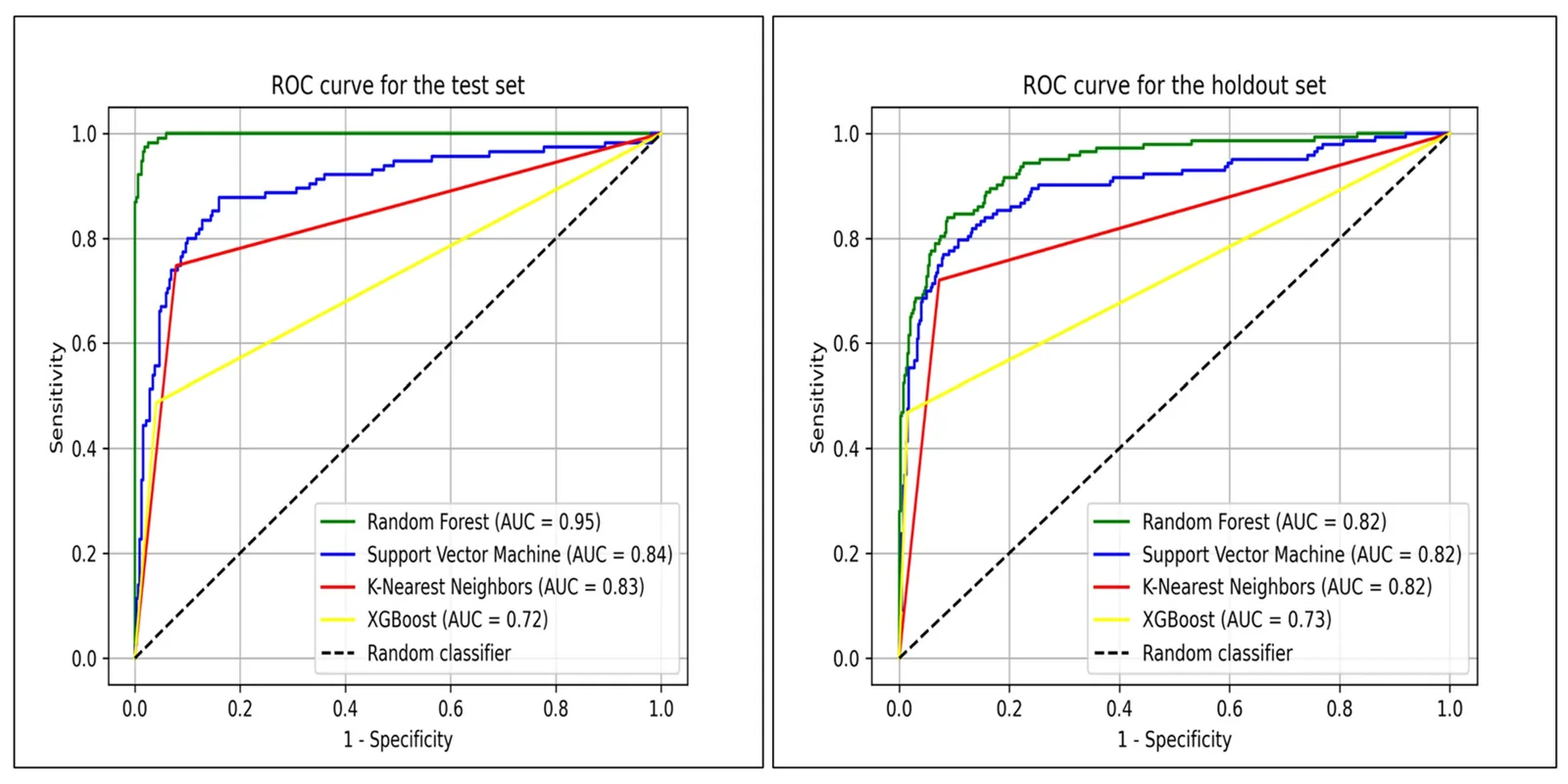
Receiver operating characteristic (ROC) curves of ML models to predict HER2 inhibitor prioritization. The test set is represented by the left panel, and the holdout set is represented by the right panel. Some of the models used are Random Forest, Support Vector Machine, K-nearest neighbors, and XGBoost.

Four machine learning classifiers, Random Forest, Support Vector Machine (SVM), K-Nearest Neighbors (KNN), and XGBoost, were tested using receiver operating characteristic (ROC) curves on test datasets and holdout datasets. On the test data, Random Forest achieved an AUC of 0.95, significantly higher than SVM (0.84) and KNN (0.83), and XGBoost got a moderate AUC of 0.72. The best predictive performance was obtained using the Random Forest model, which also gave the highest AUC values when tested on both the test and the holdout sets. However, the decrease in AUC in the test set compared to the holdout set, from 0.95 to 0.82, suggests that there may be some level of performance degradation on independent data, which is indicative of a possible decrease in a mild level of overfitting. Despite these findings, the Random Forest model had the best overall predictive performance of the classifiers tested, which is consistent with its potential as a predictor for prioritizing potential HER2 inhibitors, suggesting the need for external validation to further evaluate its generalizability. Whereas SVM and KNN showed a similar AUC of 0.82, indicating their superior generalizability. XGBoost showed only a slight improvement (of 0.73) on the holdout set and was still classed as a moderately performing model. Thus, although Random Forest performs well on the test data, the similarity of SVM and KNN on the unseen data suggests a higher degree of reliability in generalization, which highlights the need for model validation on independent holdout data sets to ensure that a model is robust and is not overestimating predictive performance, as seen in Figure 1. To provide a more comprehensive evaluation of the machine learning models, additional performance metrics including precision, recall, F1-score, Matthews correlation coefficient (MCC), and precision–recall area under the curve (PR-AUC) were calculated alongside ROC-AUC and accuracy for both the test and independent holdout datasets (Table S1).

### 2.2. Structure Preparation for Molecular Docking

In this investigation, a region of the HER2 kinase was selected as the therapeutic target because it plays a key role in the oncogenic signaling pathways in breast carcinoma [12]. The crystal structure of human HER2 (ERBB2) YVMA mutant kinase domain (PDB ID: 8U8X) was obtained from the PDB and determined by X-ray diffraction with a resolution of 1.69 Å, thus providing a very reliable and detailed atomic model suitable for structure-based drug design. Validation metrics confirm the dataset’s higher structural quality: the value of R free is 0.200, and the value of R work is 0.165, which means the agreement between experimental observations and the refined model is close. The Ramachandran analysis shows no outliers, suggesting that all backbone dihedral angles fall in favorable regions. Side-chain outliers represent only 1.1% of residues, and the RSRZ outlier fraction is 5.8%, both within acceptable outlier fractions for high-resolution crystallographic structures. The overall clash score is equal to four, which is minimal steric conflicts. Collectively, all of these validation parameters show that the 8U8X HER2 structure is of high quality and therefore fit for follow-up molecular docking and dynamic simulations. The co-crystallized inhibitor also provides a reference for defining the active site, thus leading to the precise identification of key residues that are involved in interactions with the ligand.

### 2.3. Molecular Docking Interpretation

**Figure 2:**
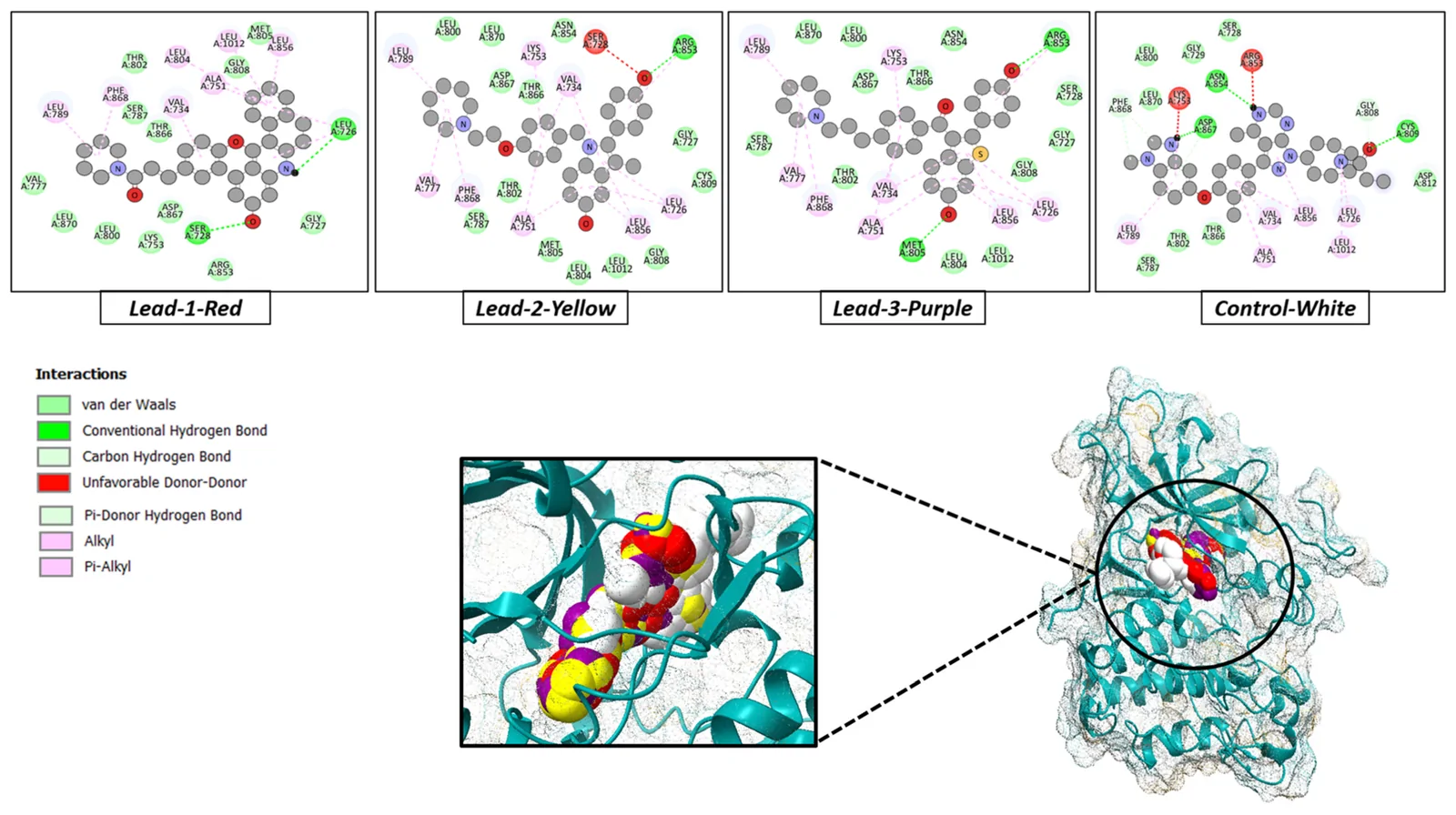
Depicts the two-dimensional (2D) (**Top**) and three-dimensional (3D) (**Bottom**) structures of HER2 with the CHEMBL193865 (Lead-1), CHEMBL46740 (Lead-2), CHEMBL151318 (Lead-3), and control. The bottom (3D) indicates the docking pose of the three compounds and references within the active site of the HER2: Lead-1 (Red), Lead-2 (Yellow), Lead-3 (Purple), and reference colors (White), respectively.

After ML-based prioritization, an active curated set of 384 compounds, including those predicted by an original ChEMBL set of 10,000 small molecules, was docked to the HER2 kinase domain. Docking analysis prioritized the top three compounds based on the high binding affinities, whereby docking scores were [−12.1 to 11.7] kcal mol, which performed better than the control compound (−10.8 kcal/mol), Table 1. Lead-1 had a slightly better docking score, but taking into account the limited discriminatory power of the docking scores, the ranking was analyzed in conjunction with the interaction profiles, molecular dynamics stability, and MM/PBSA binding free energies. The interaction analysis showed in detail that these ligands were bound to key active-site residues by several hydrogen bonds, hydrophobic contacts, and π contacts, as depicted in Figure 2. The docking poses also suggested that the novel compounds were able to bind to the ATP-binding pocket, which forms stable interactions that are vital to the mechanism of inhibition. Lead-1 was found to establish two hydrogen bonds with LEU726 and SER728 hydrophobic interactions with MET805, GLY808, THR802, SER787, THR866, GLY727, ASP867, ARG853, LYS753, LEU800, LEU870, and VAL877, which additionally stabilized the ligand in the pocket. Lead-2 showed a similar binding orientation. The redocking of the native crystallized ligand with HER2 was used to validate the docking protocol. The docked complex root-mean-squared deviation (RMSD) was then compared with the crystallized one, and an RMSD of 0.12 Å was found [13]. In sum, the docking outcomes presented a structural explanation in the selection of the ligands to be used, emphasized the existence of strong interactions of inhibition, and led to the following MD simulations in order to define dynamic stability and post-docking behavior.

### 2.4. ADME and Toxicity Prediction Analysis

All three chosen compounds and the control were then tested on the basis of drug-likeness and pharmacokinetic characteristics by ADME analysis before MD simulation. The findings showed that the selected compounds followed the Lipinski rule of five, as they had good molecular weights of less than 500 g/mol. The findings also indicated that the selected compounds had good hydrogen-bond acceptors and donors and the optimum lipophilicity, which is good for oral bioavailability. Conversely, the control compound had a borderline molecular weight and a slightly higher LogP, indicating that it might have a limitation in its absorption. Other ADME parameters, such as topological polar surface area (TPSA), solubility (LogS), and the predicted gastrointestinal (GI) absorption, added further proof that the compounds identified had good pharmacokinetic profiles. The three compounds had high predicted GI absorption and intermediate solubility, with the control compound having low predicted absorption. In general, the ADME assessment revealed that the three ranked compounds not only exhibit high predicted HER2 binding but also excellent physicochemical and pharmacokinetic characteristics of oral drug candidates, which further support their choice in downstream MD simulations and post-MD analyses. Medicinal chemistry assessment of these three lead compounds included favorable ADME properties, and none of them contained any PAINS alerts, indicating that there was no likelihood of nonspecific assay interference or false-positive biological activity. The alerts identified by Brenk analysis for Lead-1 and Lead-3 were one structural alert each (Michael acceptor and quaternary nitrogen, respectively), which is something that may need to be optimized during lead development, but does not preclude their further investigation. Interestingly, the reference compound also had a quaternary nitrogen Brenk alert. The overall medicinal chemistry profile for Lead-2 was the most favorable, which is why it is prioritized for further experimental evaluation.

The main criterion for drug likeness was chosen to be “Lipinski’s Rule of Five,” which is the most widely adopted and experimentally confirmed rule for predicting the oral bioavailability of small-molecule compounds. The aim of this study was the initial identification of lead molecules with oral activity, and Lipinski’s criteria proved to be appropriate as a first-pass evaluation of the physicochemical properties related to absorption and permeability. Other filters, such as the Veber, Ghose, Muegge, and Egan rules, provide complementary drug-likeness evaluations but are generally used as additional optimization criteria, not as alternatives to Lipinski’s rule, in early-stage virtual screening.

The toxicity profile of the chosen lead compounds was analyzed via the ProTox-3.0 web service. All three compounds were predicted to be in toxicity class 4 and have moderate acute oral toxicity with estimated oral LD50 values ranging from 500 to 1190 mg per kg of body weight (as summarized in Figures S5–S7). The lowest LD50 value was found for Compound A with 500 mg/kg, while the predicted value for tamoxifen was 1190 mg/kg and Compound C was 850 mg/kg.

From the organ toxicity predictions, all compounds were predicted to have a potential risk for neurological toxicity and respiratory toxicity, while no compounds were predicted to be cardiotoxic. Lead-1 and Lead-3 were predicted to be inactive for this endpoint. Nephrotoxicity was inactive for Lead-3 and inactive for Lead-1, and ecotoxicity was inactive for both Lead-1 and Lead-3, but active for Lead-2. The accompanying radar maps are shown to further convey the probability distribution of toxicity endpoints and interactions with toxicity-related targets, thereby giving a full visualization of the predicted safety profiles. The average toxicity profile of the identified lead compounds was similar to or better than the reference drug, which suggests they may be promising for further study.

### 2.5. pKa Prediction Findings

The predicted pKa values allowed the prediction of the protonation state of the lead compounds at physiological pH (7.4), which gave some idea of their protonation behavior and their possible effect upon protein–ligand interactions in molecular docking and MD simulations, as shown in Table 2 and Figure S7.

### 2.6. MD Simulation—Trajectory Analysis

#### 2.6.1. RMSD and RMSF

**Figure 3:**
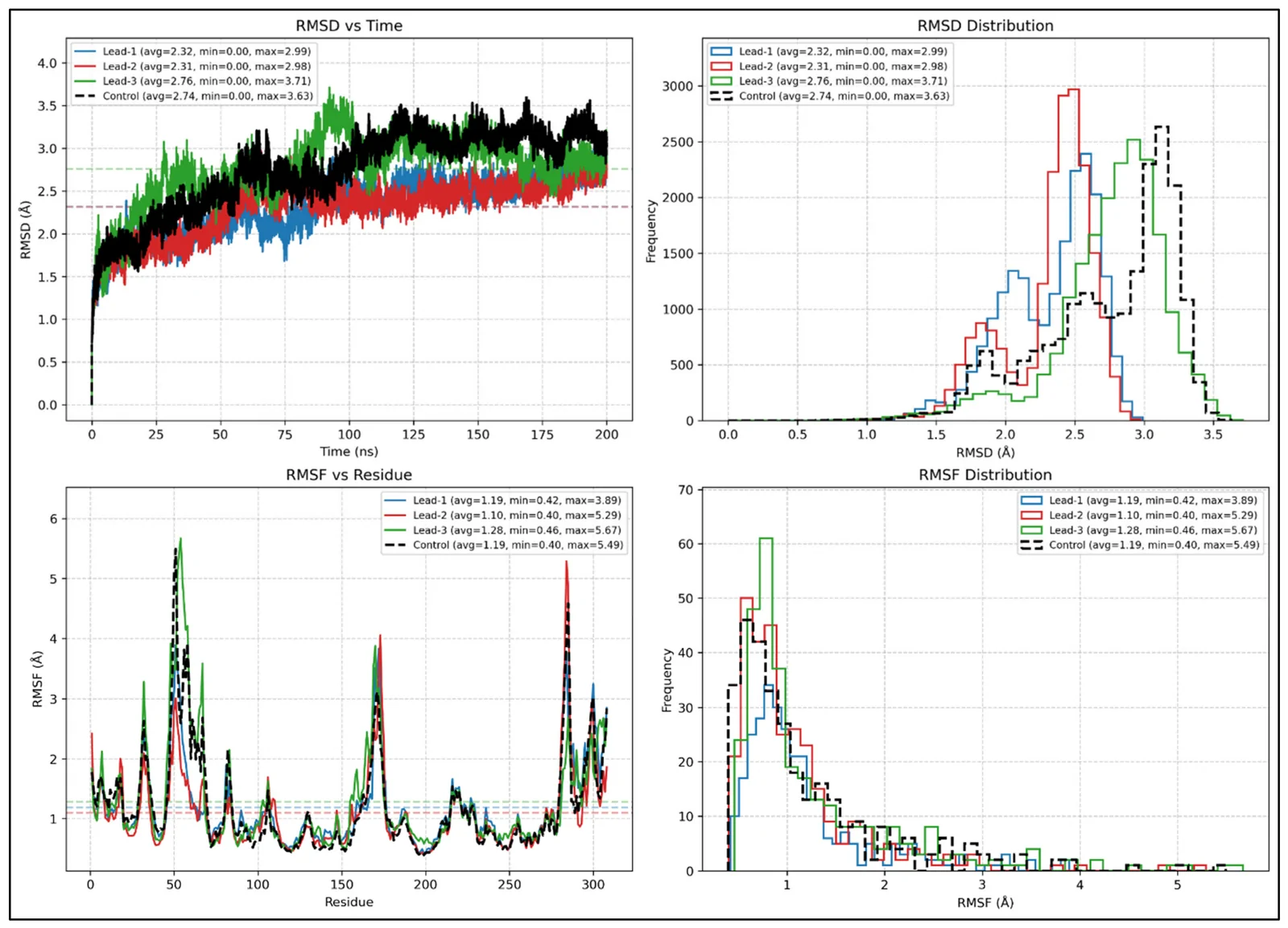
The RMSD and RMSF of HER2 in the presence of three novel ligands, CHEMBL193865 (Lead-1), CHEMBL46740 (Lead-2), CHEMBL151318 (Lead-3), and a control ligand after 200 ns of MD simulation. (**Left**): The RMSD (**top**), RMSF (**bottom**) versus time curve, while the top right shows the RMSD (**top**) and RMSF (**bottom**) distribution.

Root-mean-squared deviation (RMSD) analysis was performed to assess the structural stability of the HER2 protein in complex with three lead compounds (Lead-1, Lead-2, and Lead-3) compared to the control [14]. The plot of RMSD vs. time shows that the equilibrium of all the complexes is achieved within the first 50 ns, and then the deviations of both remain relatively constant over the course of the 200 ns simulation (Figure 3). Lead-1 and Lead-2 have lower RMSDs (2.32 Å and 2.31 Å, respectively) than Lead-3 (2.76 Å) and the control (2.74 Å), as the two leads maintain a more constant interaction with HER2 throughout the simulations. These observations are supported by the RMSD distribution plot (right), which shows that most of the conformations of Lead-1 and Lead-2 are relatively near the original structure, and Lead-3 has a few more fluctuations.

To determine the degree to which the leads would create a degree of flexibility in the protein at the residue level, root-mean-squared fluctuation (RMSF) analysis was done [15]. The RMSF vs. residue plot (left) shows that the majority of residues have low fluctuations, with clear peaks in the graph of loop regions and terminal residues, which are more flexible by nature (Figure 3). The average RMSF of Lead-2 is the lowest (1.10 Å), indicating that there is less local flexibility than in Lead-3 (1.28 Å) and the control (1.19 Å), which means that the binding of Lead-2 can provide further stabilization to important regions of HER2. These results are further supported by the RMSF plot distribution (right), in which the fluctuation of residues of Lead-1 and Lead-2 is mostly lower, but Lead-3 and the control have more flexibility in various loop regions. In general, it can be concluded that the RMSD and RMSF results indicate that Lead-1 and Lead-2 are capable of forming stable complexes with HER2, which allows them to stabilize the structures effectively and minimize their fluctuations at the residue level, whereas Lead-3 is fairly stable and more flexible. These results suggest that Lead-1 and Lead-2 have potential binding stability and can be considered for future in vitro and in vivo application.

#### 2.6.2. Rog and Beta Factor

**Figure 4:**
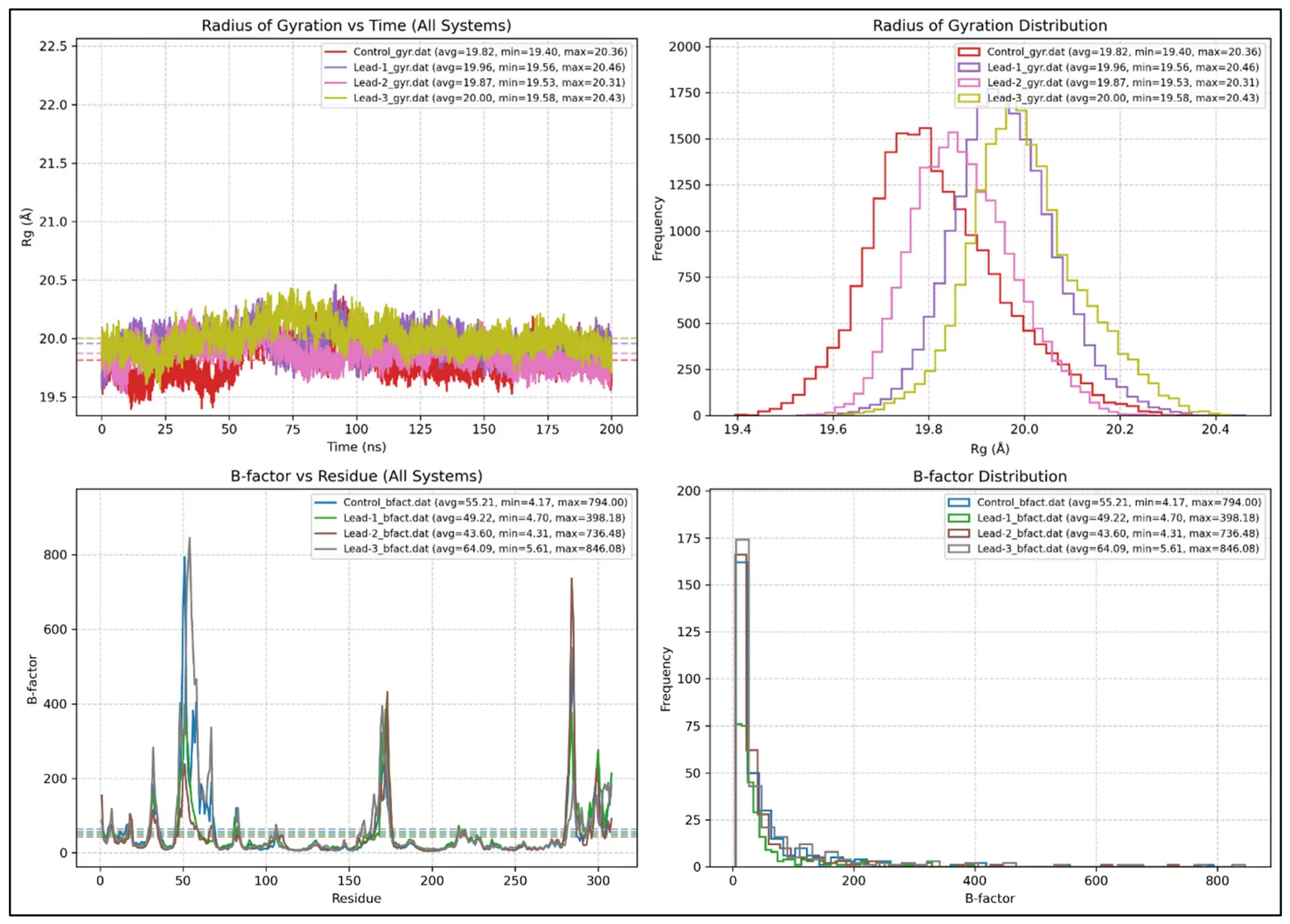
The Rog and B-Factor of HER2 in the presence of three ligands CHEMBL193865 (Lead-1), CHEMBL46740 (Lead-2), CHEMBL151318 (Lead-3), and a control ligand after 200 ns of MD simulation. (**Left**): The Rog (**top**), B-Factor (**bottom**) versus time curve, while the top right shows the Rog (**top**) and B-Factor (**bottom**) distribution.

To determine the compactness and general structural integrity of HER2 in the presence of the three lead compounds (Lead-1, Lead-2, and Lead-3) in comparison with that of the unbound control, the radius of gyration (Rog) analysis was conducted [16]. The Rog vs. time curve, Figure 4 (left), shows that there is a relatively steady Rog value in all systems within the 200 ns simulation time, implying that none of the ligands causes extensive global structural expansion or contraction. Lead-1, Lead-2, and Lead-3 have average Rog values of 19.96 Å, 19.87 Å, and 20.00 Å, respectively, a little smaller than the control (19.82 Å), which shows that there are slight changes in compactness on binding the ligands. The Rog distribution plot (right) additionally validates the results of the Rog distribution plot, showing that all of the systems sample a small range of Rog values, supporting the overall stability and maintained tertiary structure of HER2 during the simulations. It is interesting to note that Lead 2 has a slightly lower average Rog when compared to Lead 1 and Lead 3, which implies that it tends to form a smaller protein–ligand complex.

**Figure 5:**
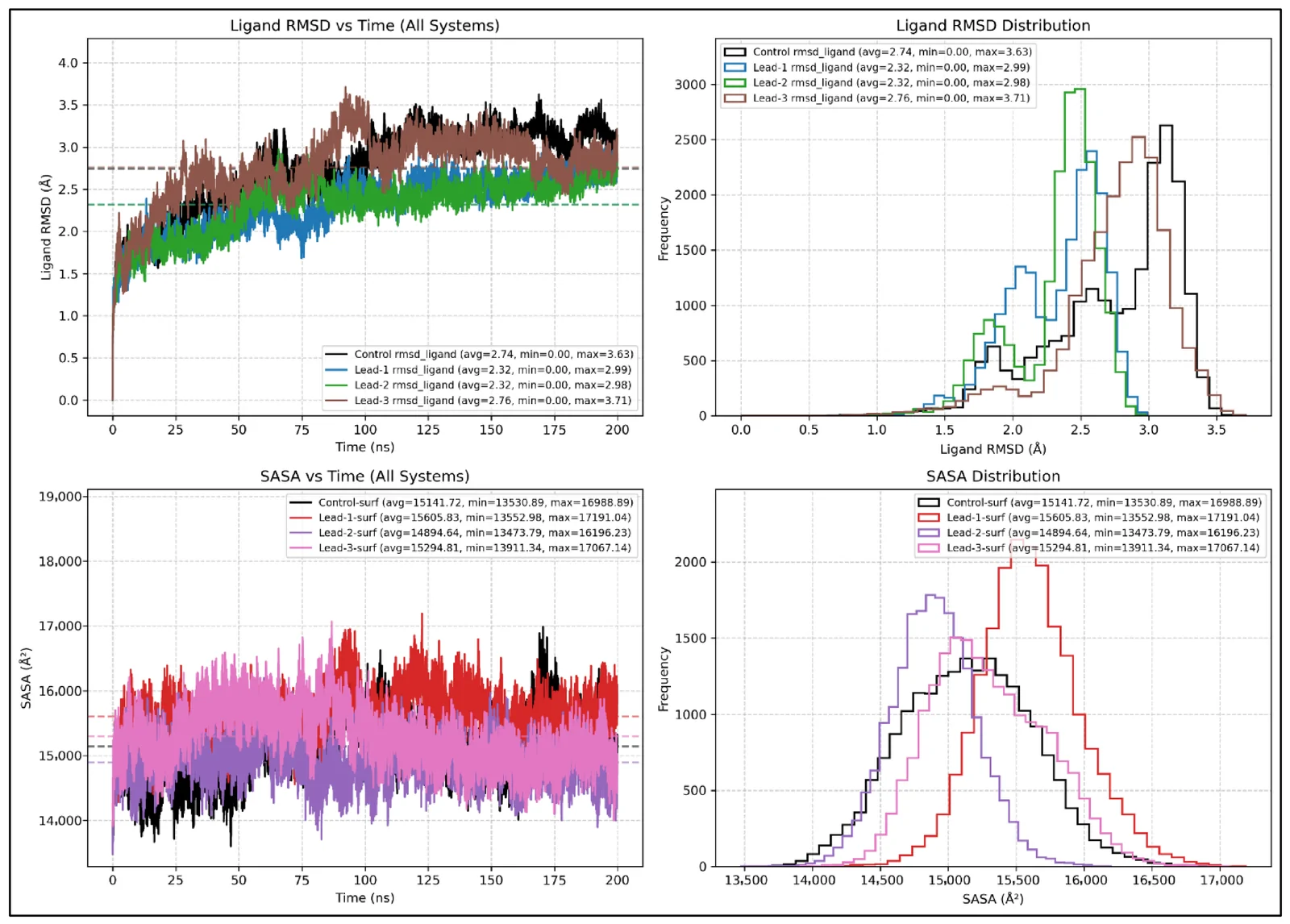
The Ligand-RMSD and SASA of HER2 in the presence of three compounds: CHEMBL193865 (Lead-1), CHEMBL46740 (Lead-2), CHEMBL151318 (Lead-3), and a control ligand after 200 ns of MD simulation. (**Left**): The Ligand-RMSD (**top**), SASA (**bottom**) versus time curve, while the top right shows the Ligand-RMSD (**top**) and SASA (**bottom**) distribution.

B-factor analysis was done to determine the residue-level flexibility and atomic displacement of all the systems [17]. The B-factor and residual plot, Figure 4 (left), indicate that the majority of the residues have low mobility, with the peaks that represent the loop regions and terminal residues being more flexible, as is expected because of inherent structure dynamics. Lead-2 has the lowest average B-factor (43.60), suggesting that it has less residue-level flexibility compared to the control (55.21) and other lead compounds, with Lead-3 having the highest average B-factor (64.09), suggesting an increase in local fluctuations. These findings are supported by the B-factor distribution plot (right), which indicates that most of the residues in the Lead-2 complex are less flexible than in Lead-1 and Lead-3, suggesting that it may have a stabilizing effect on HER2 (Figure 5). Altogether, the Rog and B-factor results reveal that all three leads support the global structural integrity of HER2, and Lead-2 is capable of providing further global and local stabilization. The results with the RMSD and RMSF indicate that Lead-2 presents a very stable and small complex with HER2, further arguing for its suitability as a promising experimental candidate.

#### 2.6.3. Ligand-RMSD and SASA

The ligand RMSD profiles show that Lead-1 and Lead-2 had lower deviations during the simulation, with an average RMSD of 2.32 Å, indicating that their binding was more stable than the control (2.74 Å) and Lead-3 (2.76 Å), Figure 5. The RMSD distribution also demonstrates that Lead-1 and Lead-2 ligands have smaller peaks and have lower maxima, which supports their stability in the binding site. On the other hand, Lead-3 appears to exhibit greater fluctuations, and the distribution of RMSD is wider, meaning that the binding is relatively unstable.

By comparing the solvent-accessible surface area (SASA) of the three systems, it is possible to see that the surface exposure of the proteins differs among the systems. The average SASA of Lead-1 (15,605.83 Å^2^) is the most significant, meaning that the solvent area is greater, and Lead-2 (14,894.64 Å^2^) has the smallest, suggesting the conformation of Lead-2 is more compact. Time-resolved SASA profiles exhibit uniform variations throughout the trajectory, which depict dynamic protein–ligand interactions (Figure 5). Collectively, RMSD and SASA analyses indicate that Lead-1 and Lead-2 ligands attain good stability in the binding pocket, but Lead-3 is characterized by high conformational variability, potentially affecting its binding affinity and the entire interaction profile. To visualize the MD simulation, the snapshots of the HER2–ligand complex at 0 ns and 200 ns were generated (Figures S9–S12). The structural stability of the protein–ligand complex was also shown, as the ligand did not undergo significant conformational changes during the course of the simulation from 200 ns, as the structure of the complex before the start and end of the simulation was compared.

#### 2.6.4. Hydrogen-Bond Analysis

The H-bond analysis indicates that there are significant variations in the stability and persistence of interactions that occur between the three new leads and the control compound during the simulation. In the case of Lead-1, the presence of many hydrogen-bond interactions was observed largely between the residues ASP99, SER17, and ARG140. The strongest and prolonged interactions were found between ASP99 (OD2 and OD1) and the ligand (LIG309) with occupancies of 10.35 and 7.94, respectively. These interactions exhibited average donor-to-acceptor distances of 2.762.77 Å and positive bond angles of 154,157 degrees, which showed that hydrogen bonding was geometrically stable. There were also minor and brief contacts with SER17 and ARG140, which have low frame fractions indicating weak interactions or short-lived interactions. Comprehensively, Lead-1 has medium and occasionally constant hydrogen bonding, mainly mediated by ASP99. Lead-2, on the contrary, has much stronger and more stable hydrogen-bond interactions. One of the most dominant hydrogen bonds is with SER74, with an occupancy of 61.21, an average distance between the donor and acceptor of 2.72, and an angle of 158.61. The strength and persistence of these contacts are supported by the short bond distances (~2.72–2.86 Å) and good bond angles, as seen in Table 3. Such observations suggest that Lead-2 forms a strong and stable network of hydrogen bonds in the binding pocket, and it is probable that this contributes significantly to the binding stability of Lead-2. In the case of Lead-3, the major interaction is between MET92, with an occupancy of 16.38 an average distance of 2.81 Å, and an angle of 148.83. In spite of this moderately stable interaction, its occupancy is significantly lower when compared to that of Lead-2. As a result, Lead-3 exhibits moderate hydrogen-bond stability with no persistent strong interaction with the molecule, as is the case with Lead-2. The control compound has a general weak interaction of a hydrogen bond. The presence of contacts with TYR94 and ARG102 is at very low occupancy (~0.4), though bond distances of about 2.882.95 and angles of 138,153 are favorable. This small persistence of these hydrogen bonds indicates that these hydrogen bonds do not form well in the binding site. Overall, the occupancy of the hydrogen bonds and the geometrical parameters show that the hydrogen-bonding profile in Lead-2 is the most stable and strongest, in order of Lead-3 and Lead-1. These results indicate that Lead-2 probably has better binding stability, owing to the continued and geometrically favorable hydrogen-bond interactions that occur in the active site.

#### 2.6.5. PCA and FEL

**Figure 6:**
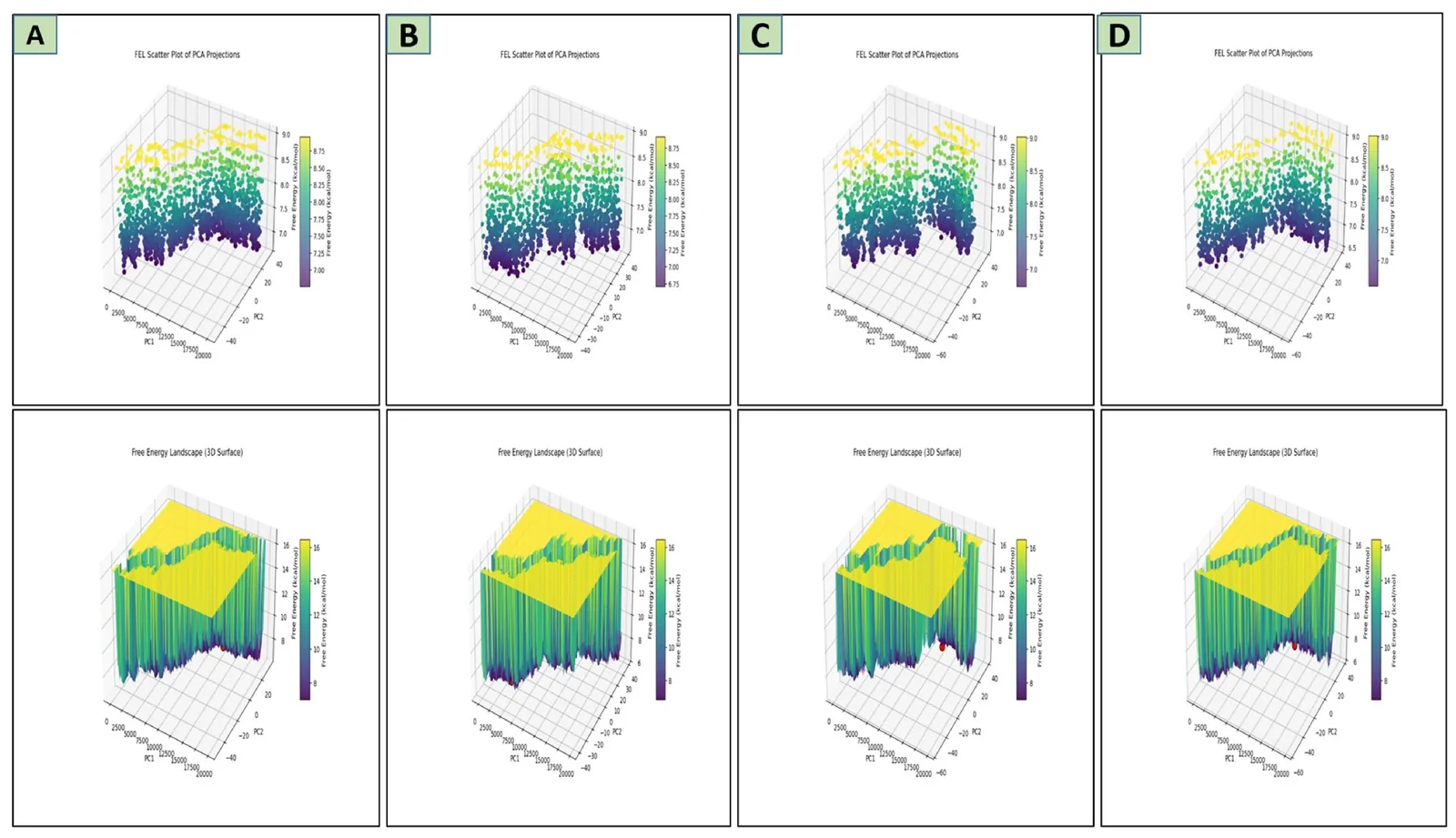
Depicts the PCA and FEL of the HER2–ligand complexes in the course of the MD simulations. The conformational sampling and dominant motions of three novel compounds, CHEMBL193865 (**A**), CHEMBL46740 (**B**), CHEMBL151318 (**C**), and the control complex (**D**), are shown.

The free-energy landscape (FEL) is a computational and thermodynamic model that is used to describe all of the possible conformational states of a molecular system, including a protein–ligand complex, and their corresponding free-energy values. It gives a topographical map, which shows system stability and transitions, with low-energy regions representing mechanically stable conformations and high-energy regions representing less favorable or unstable ones [18]. Principal component analysis (PCA) is a statistical analysis method that will decrease the size of large datasets and keep the most important patterns of variance [19]. In MD simulations, PCA is used to represent the atomic coordinates to highlight major molecular movements. As depicted in Figure 6, the three novel complexes (A, B, C) and the control compound (D) were investigated using the FEL scatter plots (top row) to determine their conformational stability and energy distribution. Complexes A, B, and C were seen to have dense, well-distributed clusters of conformations over the major components, with color gradients depicting free-energy changes in the order of about 7 to 9 kcal/mol/−1. These data suggest that the complexes have the ability to sample multiple conformational states with a comparatively low energy, which is a characteristic of stable binding behavior. Complex B showed a more diffuse distribution on PC2, which hinted at a slight degree of flexibility compared to complexes A and C. These distinctions were further explained using the three-dimensional free-energy surfaces (bottom row). The systems preferred deep and well-defined energy basins in complexes A, B, and C. On the other hand, the control compound (D) had a relatively less deep, rugged topography, indicating a lack of conformational energy and an increase in temperature fluctuations. Taken together, these analyses point to the fact that the three novel complexes are in energetically favorable, stable conformations with relatively small differences in the flexibility profile, whereas the control compound is not characterized by structural stability, indicating the potential of the novel complexes as promising candidates with favorable thermodynamic and conformational characteristics.

#### 2.6.6. DCCM

**Figure 7:**
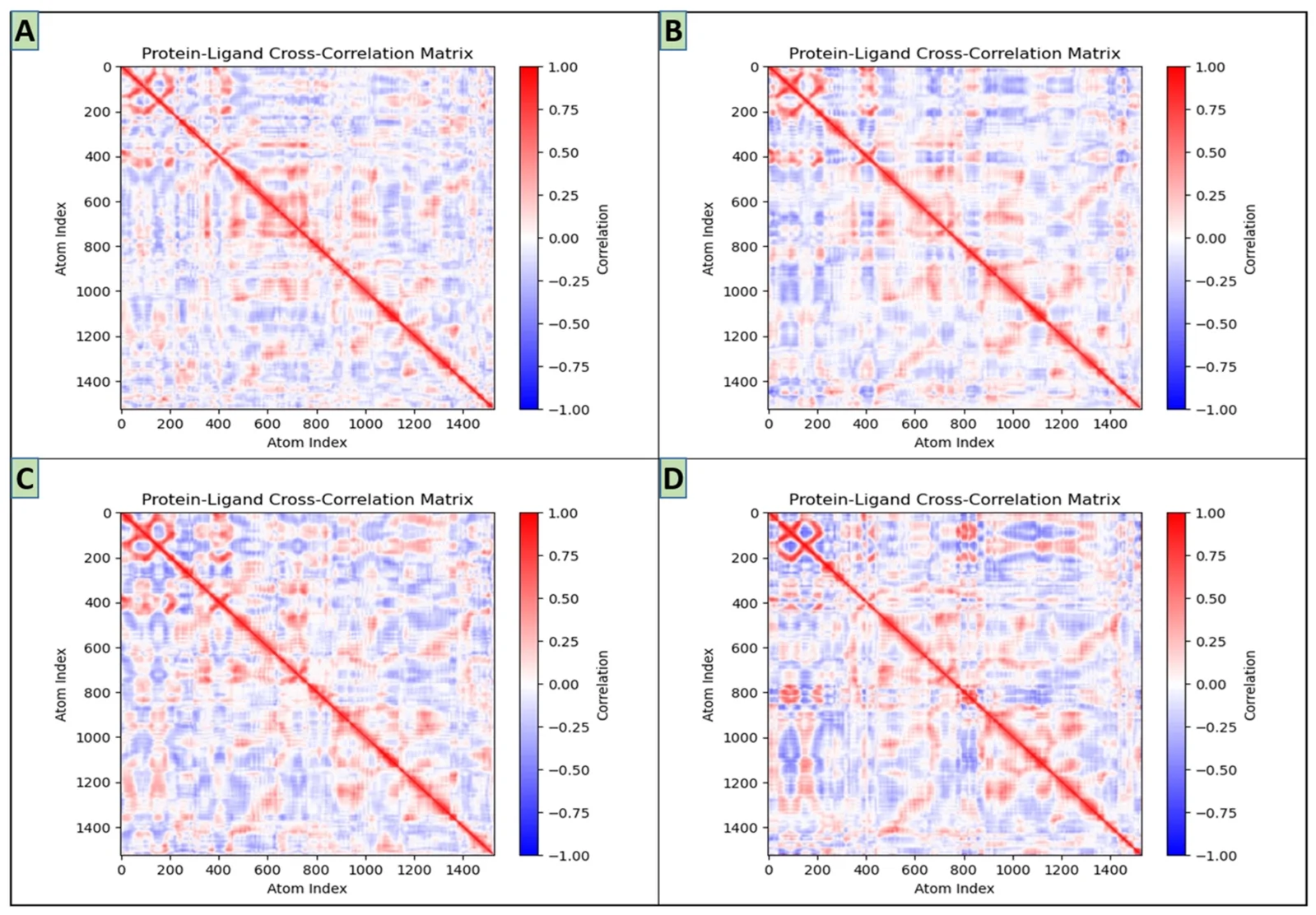
Exhibits the DCCM of the HER2 kinase domain in complex with compounds CHEMBL193865 (**A**), CHEMBL46740 (**B**), CHEMBL151318 (**C**), and control (**D**) during the course of the simulation. The positive (correlated) and negative (anti-correlated) motions of the residues are denoted, explaining the effect of each ligand on residue-residue interaction and dynamics.

Dynamic cross-correlation matrices (DCCM) are calculation methods used regularly in MD simulations to assess correlated motions between residues or atoms within a protein or other biomolecular system [20]. The protein–ligand cross-correlation matrices (CCM) of the four complexes (A, B, C, and D) indicate that the protein atoms have specific dynamic behaviors in response to the binding of the ligand. The correlation of the motion between pairs of atoms is plotted in each of the matrices, where the value is −1 (completely anti-correlated, shown in blue) to +1 (completely correlated, shown in red), Figure 7. The matrix of complex A shows moderate to strong positive correlations over the diagonal, which indicates coordinated movement of neighboring atoms, and scattered blue areas, which indicate localized anti-correlated motions that could represent subtle conformational changes on ligand binding. Complex B exhibits a slightly less pronounced positive correlation pattern, and the red patterns are more diffuse, and the anti-correlated motions are more pronounced, in comparison with A, suggesting it has more flexible or less-synchronized dynamics in response to its ligand. Complex C exhibits a combination of correlated and anti-correlated motions, where distinct clusters of positive correlations are observed along the diagonal, which is evidence of stable regions among flexible regions that are influenced by the ligand in specific areas of the protein. Conversely, the control compound (D) is the most anti-correlated (especially off-diagonal) and has the strongest correlated clusters on the diagonal, i.e., some structural regions might be stable, others anti-correlated in their motion, and this may indicate weaker or non-specific binding effects. In general, these cross-correlation patterns indicate that the novel ligands (A–C) stabilize the dynamics of specific regions of the proteins and allow flexibility in other regions, whereas the control (D) stabilizes the dynamics of the specific intercalants in the control, stabilizing the specificity and potential of the novel compounds.

#### 2.6.7. RDF Studies

**Figure 8:**
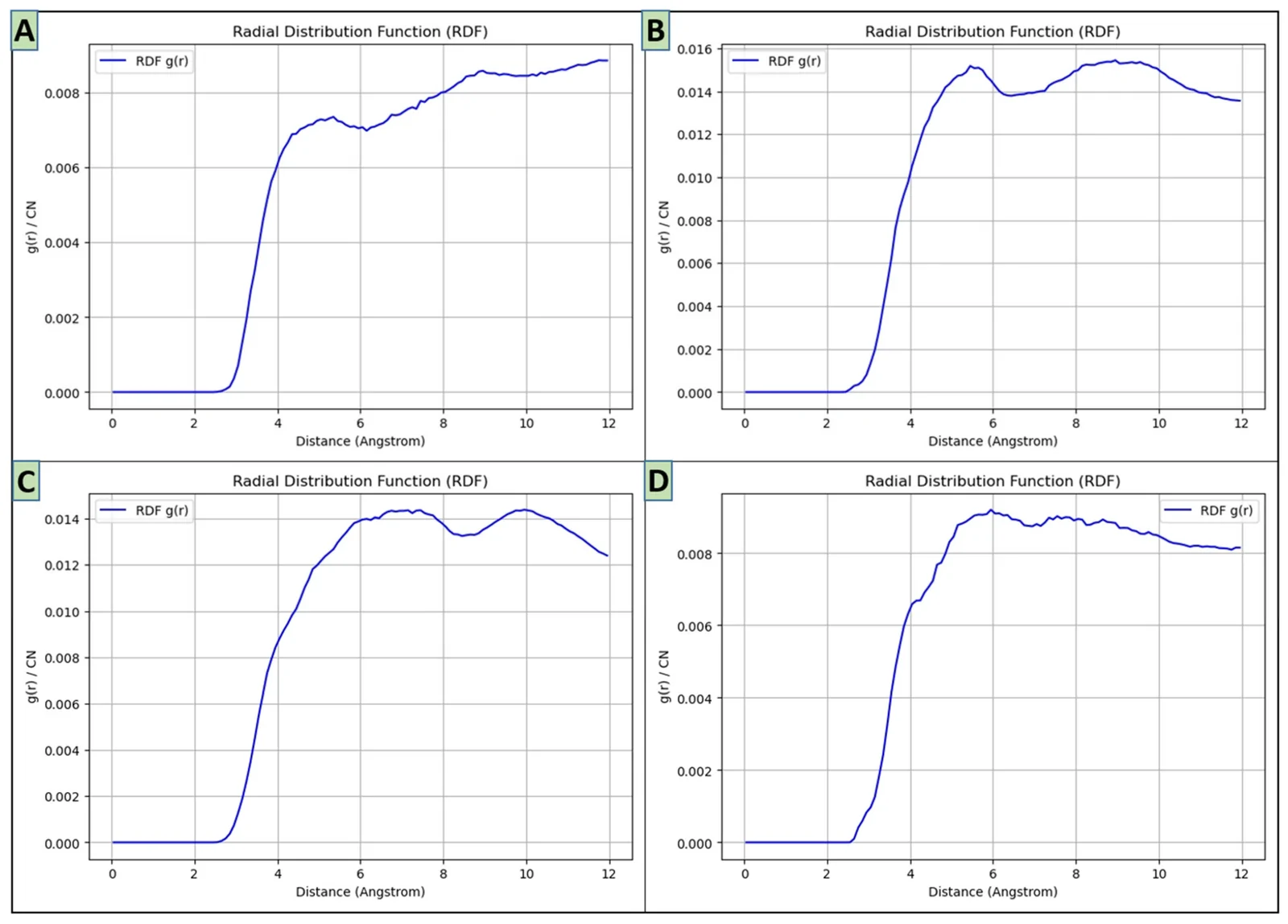
Shows the RDF interaction profile between ligands and proteins—HER2 complex with compound CHEMBL193865 (**A**), CHEMBL46740 (**B**), CHEMBL151318 (**C**), and control (**D**). The position of the peaks illustrates the preferred distances between the ligand atoms and select residues in the binding pocket during the molecular dynamics simulations.

A radial distribution function (RDF) was carried out to investigate the local structural organization and intermolecular interactions in the new complexes (A, B, C) and control compound (D) [21]. The RDF measures the likelihood of finding a particle at a certain distance if a particle is in the presence of a reference particle, thus giving an understanding of the way atoms are packed and how they interact. From Figure 8, in the case of complex A, the RDF plot shows a major peak in the range of 4–10, signifying a medium level of short-to-long-range order, then gradually rising in value, suggesting less pronounced long-range interactions. Complex B displays a more intense, high peak at 5:6 Å, which predicts more intense and ordered interactions between atoms, and there is also an additional shoulder indicating secondary coordination or hydration layers. Complex C has specific primary and secondary peaks of 6–8 Å, in evidence of a well-organized interaction network and possibly greater stability in the complex environment. Conversely, the control compound D has a single and broad peak with a center close to 5–6 Å, and is indicative of weaker or less structured atomic interactions compared to the new complexes. Overall, the RDF profiles show that complexes B and C have higher levels of organized atomic structures and stronger intermolecular forces than complex A and the control, which is associated with greater stability and positive binding properties in a biological setting.

#### 2.6.8. Secondary Structure (SS) Analysis

**Figure 9:**
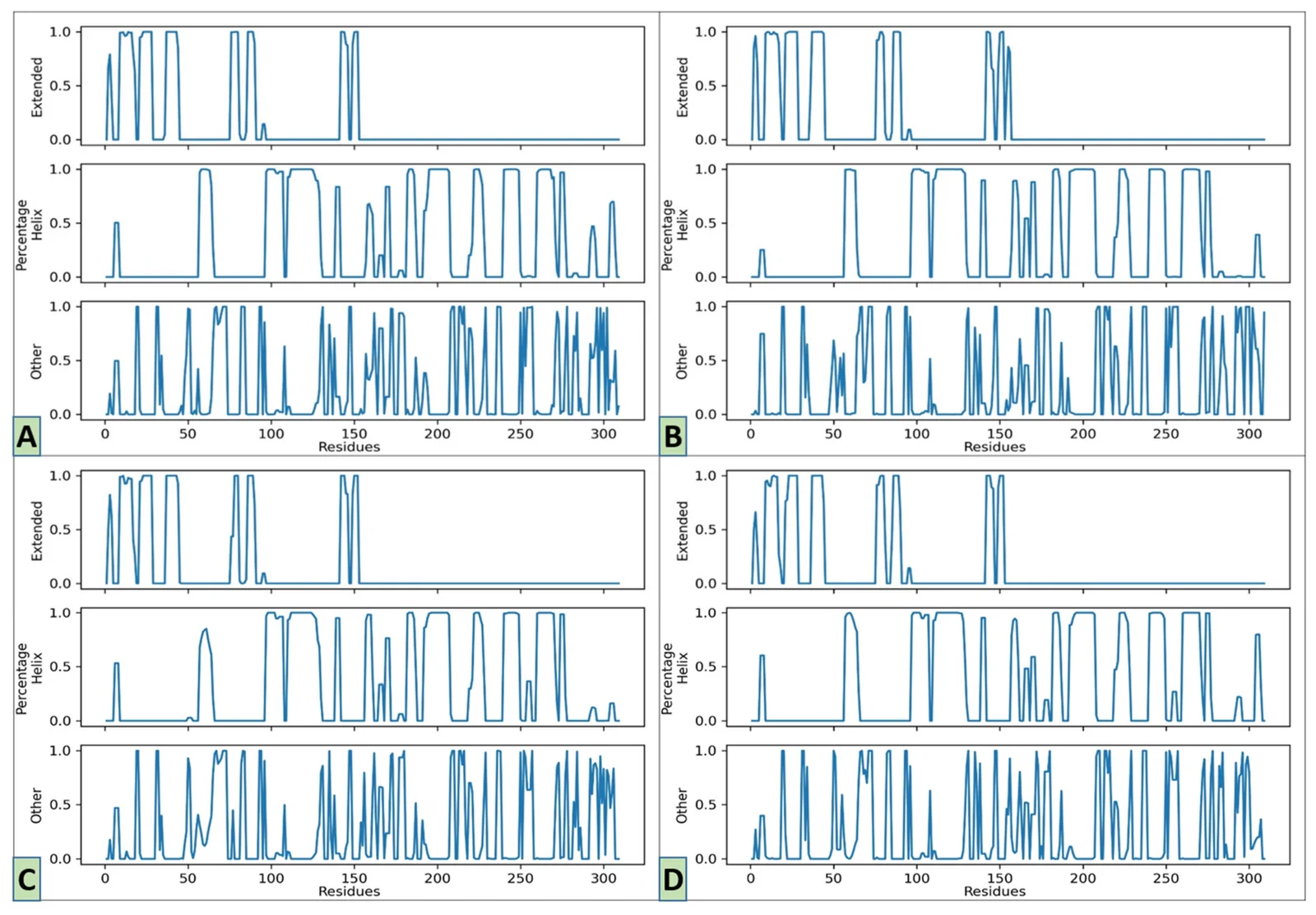
Depicts the evolution of HER2 kinase domain secondary structure in complex with compounds CHEMBL193865 (**A**), CHEMBL46740 (**B**), CHEMBL151318 (**C**), and control (**D**) throughout MD simulation.

This figure illustrates the distribution of the per-residue secondary structure of the protein with three novel ligands (A, B, and C) and a control ligand (D), as observed during trajectories of MD simulation, Figure 9. The panels depict the presence of prolonged (β-sheet), alpha-helical, and non-regular (turns/coils) constituents of the 1310 sequence of residues, thus highlighting the structural stability and conformational changes that might have taken place during ligand binding [22]. The β-sheet areas are more stable in the N-terminal region, and α-helical regions are quite stable in the central and C-terminal regions of complex A. There are small changes to coil conformations that imply conservation of the natural fold. Complex B maintains the general secondary structural pattern but has slightly more fluctuations in individual helical domains, which is suggestive of moderate flexibility. Complex C exhibits a strong retention of both sheet and helix, and a less irregular transition, thus implying greater structural stability compared to complexes A and B. Control complex D, on the other hand, has comparatively larger fluctuations, particularly in helical regions, which occasionally change to coil forms. Even though the global fold is not destroyed, the higher numbers of non-regular structures represent relatively lower stability. In general, without global unfolding, all systems maintain the basic secondary structure. However, the new complexes, especially C, followed by complex A, show much better maintenance of secondary structural features as compared to the control, suggesting increased conformational stability on the binding of the ligand.

#### 2.6.9. Electrostatic Interaction Studies

**Figure 10:**
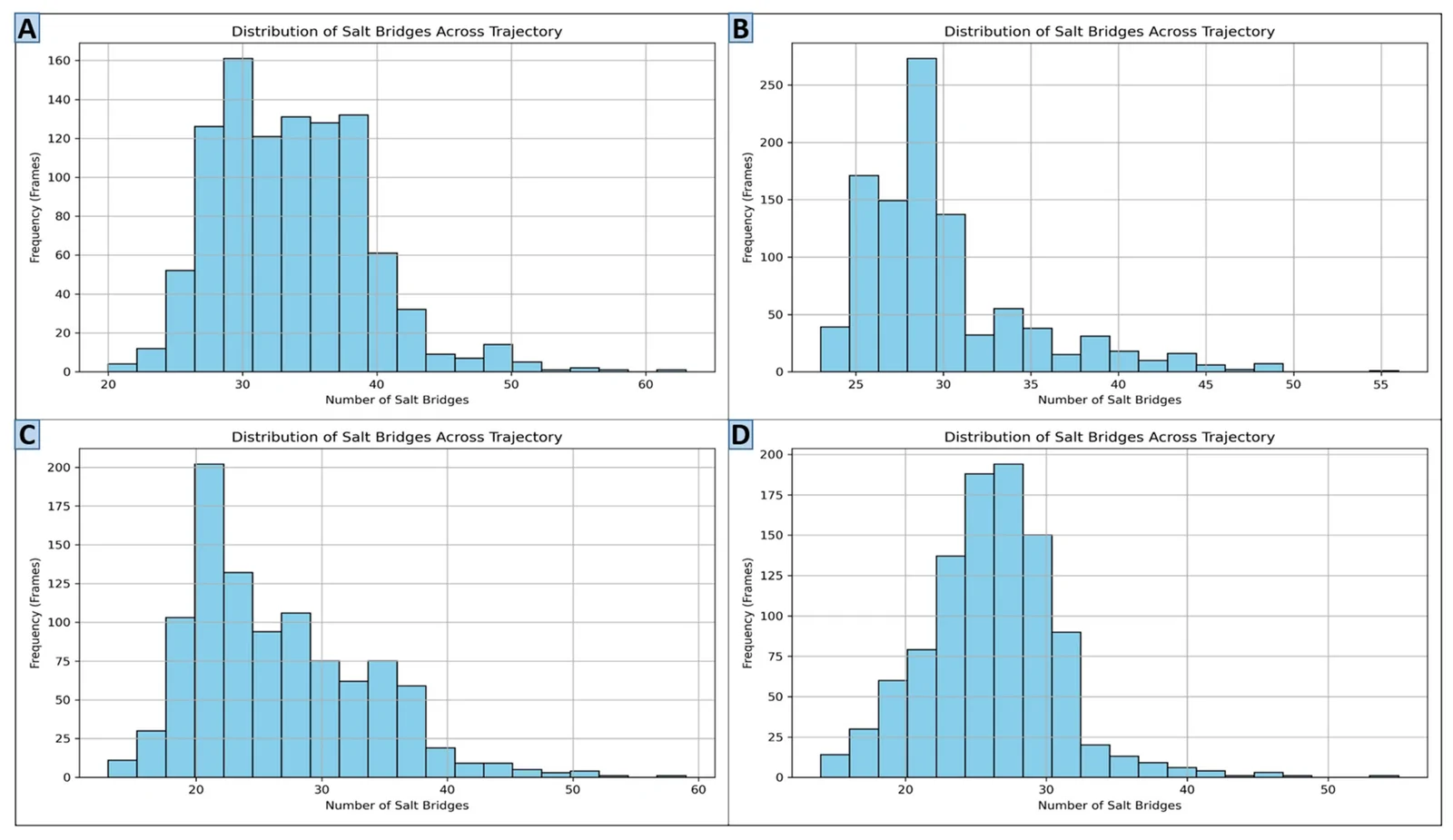
Salt-bridge analysis of HER2–ligand complexes as Lead-1 (**A**), Lead-2 (**B**), Lead-3 (**C**) and Control (**D**), formed between the charged residues of the kinase domain and functional groups of the ligands versus simulation time is plotted.

Figure 10 shows how the salt bridges are distributed throughout the MD trajectories of the three new complexes (A–C) as well as the control complex (D). The histograms represent the frequency of the simulation frames with a specific number of salt bridges and thus show the stability and the persistence of the electrostatic interactions during the simulation. In the case of complex A, the count of salt bridges has a wide distribution, with most of the frames having 30–40 interactions in the mean. The comparatively broad distribution and the sometimes high concentration (>45) are evidence of dynamic and consistently occupied electrostatic contacts, which implies the presence of constant inter- and intramolecular ionic interactions during the course [23]. Complex B has a slightly less distributed pattern and does not contain many high-value outliers, most of them falling in the range of 25 to 32 salt bridges. This indicates relative stability, and the salt-bridge formation is rather steady but a little bit smaller in number than complex A. The fact that the higher counts are occasional (as counts 40–50) suggests that the electrostatic networks were reinforced intermittently. In complex C, there is a small skew to the lower end of the distribution, but with the majority of the distribution between 20 and 30 salt bridges, although counts are occasionally observed to be higher. Although the system maintains constant ionic interactions, the relatively low average indicates a relatively elastic electrostatic network when compared to complexes A and B. The distribution of the control complex D is predominantly in 22 to 30 salt bridges, with less extreme numbers, and is more symmetrical. Salt bridges are always formed, but their total increase does not appear as significant and dynamically stronger as in the case of complex A. Collectively, Complex A has the richest salt-bridge population, which means that electrostatic stabilization is stronger and more dynamic. Complexes B and C are moderately stable, with complex C having fewer and less variable salt-bridge interactions than control complex D. These results indicate that the simulated protein electrostatic stabilization is enhanced by the novel compounds, especially A, during the simulation.

#### 2.6.10. MM-PBSA Analysis

The MM/PBSA binding free-energy computations provide a complete measure of the energetic determinants of the stability of protein–ligand complexes. It was noted that Lead-1 showed the best total binding free energy of (−107.3 kcal mol), followed by Lead-2 (−97.84 kcal mol), Lead-3 (−93.98 kcal mol), and the control compound (−86.06 kcal mol), and thus, the most favorable binding affinity of the lead compounds was observed compared to the reference. This binding energy was mostly due to van der Waals forces, with Lead-1 showing the strongest stabilization (−105.36 kcal mol), indicating the existence of strong hydrophobic interactions at the active site. All the complexes were also favorable with regard to the electrostatic interactions, especially with Lead-3, the binding strength of which was also weak due to a high polar solvation energy, Table 4. Generally, the higher total energy of Lead-1 is due to a balanced combination of both strong van der Waals and electrostatic interactions with a relatively lower desolvation energy, thus highlighting it as the most stable and most promising of all the selected compounds.

## 3. Discussion

Breast cancer, especially HER2, is still a significant health burden in the world because of its aggressive disease progression and resistance to standard therapeutic interventions [24]. The small-molecule inhibitors that target the kinase domain of HER2 have become one of the promising options in order to slow tumor development and improve clinical outcomes. We combined ML-directed compound prioritization, molecular docking, and molecular dynamics (MD) simulations in the current study to screen putative HER2 compounds systematically.

In the first phase, an interface based on machine learning (ML) was used to rank compounds obtained from a large chemical library (ChEMBL) based on their predicted activity profiles. With the initial application of ML, the chemical space was effectively reduced, and thus, a better accuracy of the candidate selection was achieved in comparison with the conventional workflow that begins with docking. Analogous ML-based prioritization algorithms have also been published in the recent HER2 computational literature, with QSAR models and pharmacophore mapping used to identify lead compounds with low-micromolar inhibitory potential (e.g., IC50 values in the low micromolar range) in large public databases [25].

The binding affinities and interaction motifs between HER2 and the ML-selected candidates were also studied in some detail in subsequent molecular docking analyses. The outcome of docking indicated that the most active compounds stabilized themselves at the active site of HER2 and formed stable interactions with the essential residues, such as hydrogen-bond networks and hydrophobic contacts, which are essential to the inhibitory activity. The docking study prioritized three compounds: CHEMBL193865 (Lead-1), CHEMBL46740 (Lead-2), and CHEMBL151318 (Lead-3), based on their binding affinity score, ranging from 12.1 to 11.7 kcal/mol. These findings come in line with other past in silico HER2 studies in which virtual screening and docking identified phytochemicals with strong affinity and good binding affinity [26]. In order to test the dynamic stability of the identified complexes under physiologically relevant conditions, 200 ns MD simulations were performed. Any trajectory measurements, such as RMSD, RMSF, radius of gyration, and hydrogen-bond persistence, revealed that the best complexes were able to keep their structures intact over the course of the simulation. This corroboration was reinforced, and results are consistent with other HER2 computational studies in which MD validated occupancy of the natural product hits and small-molecule leads in the HER2 pocket sustained over longer periods of simulation [27,28]. A good interaction pattern has been found for the selected lead compounds, which means that they can form stable interactions in the ATP-binding pocket of the HER2 receptor. The hydrogen-bond stabilization, hydrophobic interactions, and electrostatic interactions with catalytically important residues were observed during all MD simulations. These interactions have been shown to be important elements in the maintenance of kinase inhibition through a decrease in the mobility of the ligand and an increase in binding specificity. Moreover, the consistently good MM/PBSA binding free-energy values indicate the formation of energetically stable complexes under physiologic conditions. All these results suggest that the identified compounds have structural properties that enable good predicted inhibition of HER2 and thus require more experimental investigation. The HER2 kinase domain has several tyrosine kinase inhibitors (TKIs) that are effective in treating breast cancer, such as lapatinib, nelitinib, tucatinib, pyrotinib, and afatinib, which bind to the ATP-binding pocket of the kinase. Molecular docking, MD simulations, and binding free-energy calculations are also recent methods used in computational studies to discover new HER2 inhibitors, with the reference compounds being the approved drugs [29]. Just like these successful inhibitors, the compounds identified in the present study were found to be stable and to interact with the HER2 binding site throughout the MD simulations with favorable structural stability. In contrast to previous studies that focused mainly on virtual screening or structural optimization of known HER2 inhibitor scaffolds, the present work used an integrated computational framework that included virtual screening using machine learning, molecular docking, MD simulations, MM-PBSA binding free-energy calculations, and extensive ADME evaluation for prioritization of candidate molecules. This multi-tiered approach allowed the identification of structurally distinct molecules that had a good predicted binding affinity, stability, and pharmacokinetics.

The importance of hybrid workflows in modern drug discovery is explained by the integration of various layers of computation. As an example, high-throughput screening, docking, and MD have been used together with other research efforts to nominate promising HER2 inhibitors among large collections of compounds, thereby increasing enrichment and predictive power. In comparison with these works, our pipeline is the only one to focus on ML as early as possible, thus enhancing the performance of the filtering process prior to structural analysis. Not only does this strategy save on computational space, but it also enhances the likelihood of discovering actually active molecules in large chemical spaces.

While the integrated computational approach used in this study offers a complete guide for the identification of potential HER2 inhibitors, a few limitations in this approach should be noted. First, all conclusions are drawn from purely computational methods, such as machine learning, molecular docking, MD simulations, molecular-binding free-energy calculations (MM/PBSA), and in silico ADMET prediction methods. These methods can help a lot to gain confidence in lead prioritization, but do not fully reproduce the complexity of biological systems. Therefore, binding affinities, structural stability, pharmacokinetic properties, and biological activities predicted from these studies need to be tested in experiments such as biochemical enzyme inhibition assays, cell-based studies, and, finally, in vivo studies. Moreover, while the computational process worked well to prioritize promising lead compounds, further medicinal chemistry optimization could be necessary to enhance potency, selectivity and drug-like properties for clinical development. The compounds identified as leads should not be considered confirmed HER2 inhibitors and should be treated as interesting candidates for experimental evaluation in the future by computer-aided design.

## 4. Methodology

**Figure 11:**
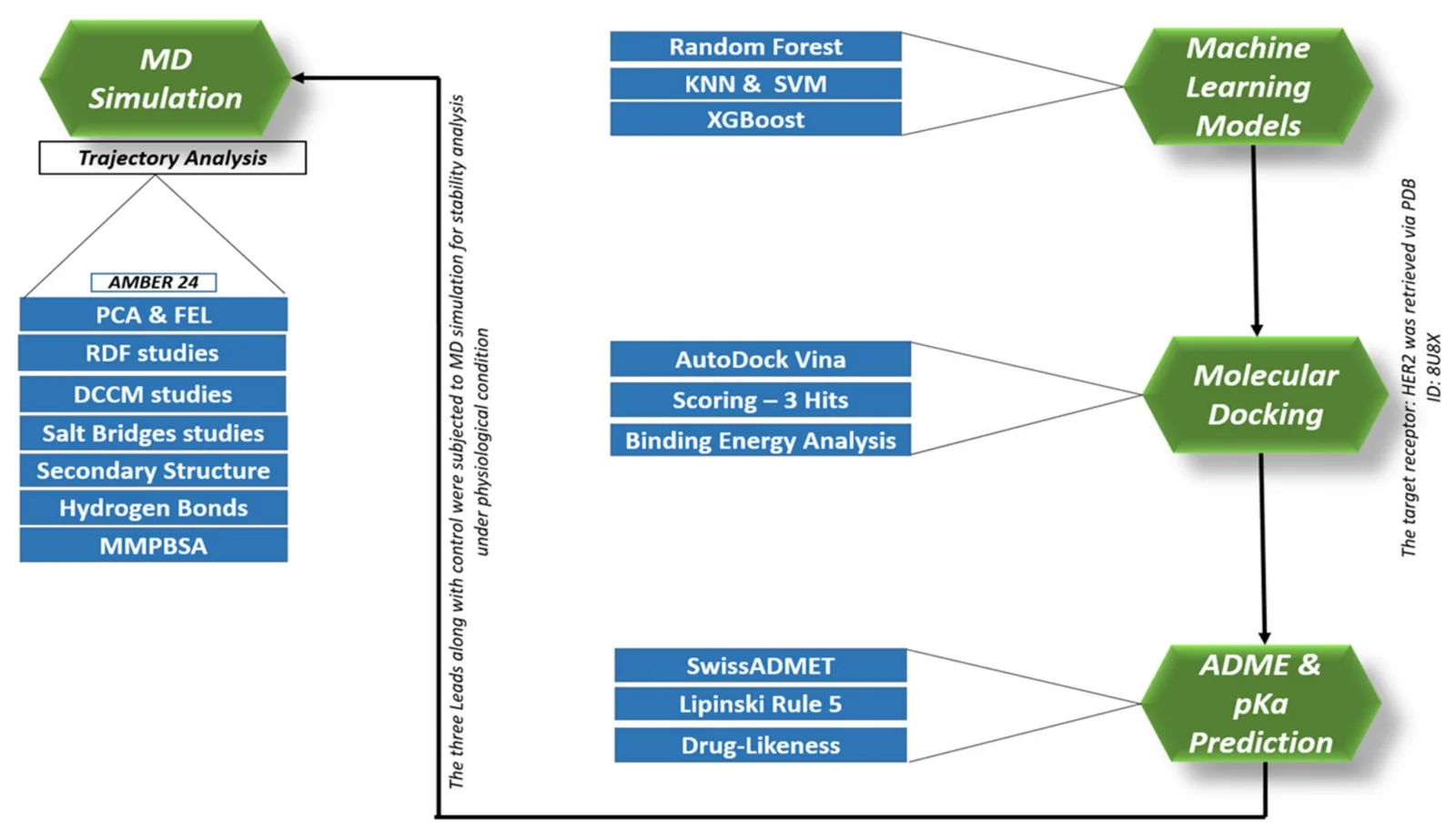
Illustrates the overall methodology involved in the current study. The study was initiated with the ML study for the identification of active and inactive compounds. The active compounds were tested in molecular docking; afterwards, the hits were identified. The ADMET and pKa prediction was carried out. Subsequently, the MD simulation was performed. Post-MD analyses were carried out, such as PCA, FEL, RDF, DCCM, salt bridges, secondary structure, hydrogen bonds, and MMPBSA.

Figure 11 depicts the methodology of the current study.

### 4.1. Data Collection and Data Curation

A systematically curated dataset consisting of bioactive compounds that were reported to modulate the activity of HER2 (Human Epidermal Growth Factor Receptor 2) was obtained from the ChEMBL database. Duplicate SMILES entries have been cleaned to maintain dataset integrity. For compounds with more than one reported value for the amount of activity, the lowest IC50 value was reported to reflect the most potent activity [30]. In total, 10,000 compounds were put together. Among this set of compounds, 384 were grouped as experimentally active, while the rest were labeled as inactive based on known activity cutoffs. Binary labeling of each compound based on compound activity (active = 1, inactive = 0) was performed for supervised machine learning analysis [31].

### 4.2. Descriptor Generation and Features Processing

The curated data was imported into Python 3.7 and preprocessed in Pandas. The molecular structures were passed to the model through molecular SMILES strings, which were converted to numerical features using RDKit. Each compound was described by a combination of molecular descriptors, such as physicochemical and topological descriptors [32]. Additionally, Morgan circular fingerprints (ECFP-like fingerprints) with radius 2 and fingerprint length 2048 bits were calculated, representing a fuller description of molecular substructures related to HER2 inhibitory activity. Duplicate entries and invalid molecular structures were first eliminated before model development. The dataset was split into a training and independent testing set with an 80:20 train–test split to maintain class balance. The feature matrix obtained was formed by molecular descriptors along with Morgan Fingerprint vectors, and the target variable was coded as binary class labels (active = 1, inactive = 0) [33].

### 4.3. Model Validation and Hyperparameter Optimization

To reduce the sampling bias and enhance the robustness of the model, the performance of the model was assessed with a stratified 5-fold cross-validation approach in the training set. Hyperparameter optimization was done using the GridSearchCV method of Scikit-learn [34]. A handful of different combinations of model-specific hyperparameters were tested for each algorithm, and the set of hyperparameters that best cross-validated the model was chosen as the final model. Optimized models were then tested on the independent test set with respect to accuracy, precision, recall, F1 score, Matthews correlation coefficient (MCC), receiver operating characteristic (ROC), and area under the ROC curve (ROC-AUC). These complementary measures were used to give a full evaluation of the predictive performance of each of the classifiers [35].

### 4.4. Development and Training of a Machine Learning Model

(a) **K-Nearest Neighbors (KNN)**

The KNN classifier was created using the KNeighborsClassifier from Scikit-learn. Various values of k (1–10) were analyzed to determine the best neighborhood size for classification [36].

(b) **Support Vector Machine (SVM)**

Both linear and non-linear SVM models were trained using Scikit-learn’s SVC. The value of the gamma parameter was set to “scale,” and hyperparameters of the linear and radial basis function (RBF) kernels were optimized in order to increase the predictive behavior [37].

(c) **Random Forest (RF)**

Random Forest classifiers were implemented in Scikit-learn using Scikit-learn’s RandomForestClassifier. This ensemble approach creates multiple decision trees and combines predictions of these trees by majority voting, which will lead to better generalization and decreased overfitting [38].

(d) **XGBoost**

An XGBoost classifier was then used using a framework of gradient boosting in which a series of decision trees are developed to minimize the prediction errors. There were some intrinsic regularization mechanisms for better predictive robustness and reduced overfitting. Compounds exhibiting probabilities of activity: > 3 > 2 > 1 > 0 (strong origin). Compounds with predicted activity probabilities ≥0.80 were prioritized for further molecular docking studies to proceed with the design of high-confidence HER2 inhibitor candidates [39].

### 4.5. Target Identification/Preparation

HER2 is a receptor tyrosine kinase that has been implicated in the progression and metastasis of breast cancer. The choice of HER2 as a therapeutic target was made considering the well-established role in the oncogenic signaling pathway and clinical relevance of HER2 in HER2-positive tumors [40]. The three-dimensional crystal structure of HER2 was downloaded from the Protein Data Bank (PDB). For molecular docking and MD simulations, the crystal structure of the HER2 kinase domain (PDB ID: 8U8X) was chosen because it offers a high-quality experimental model of the HER2 kinase domain, which has a well-defined active site and a co-crystallized ligand that allowed the test of the docking protocol by redocking and RMSD analysis. Protein preparation included the removal of water molecules, ions, and co-crystallized ligands, except if they were used for validation purposes. Missing residues were modeled if required, hydrogen atoms were added, and appropriate protonation states were assigned at physiological pH. Energy minimization was done to eliminate steric clashes and to optimize the geometry of the protein before starting the docking studies [41]. The binding pocket was defined according to the coordinates of the co-crystallized ligand and assigned by active site residue information from earlier structural studies.

### 4.6. Molecular Docking Analysis

Compounds that were prioritized from the machine learning screening phase were then submitted to molecular docking to determine the binding affinity towards HER2. Ligand structures were converted from SMILES strings to three-dimensional conformations and followed by energy minimization to get stable conformations [42]. Docking was performed by a suitable docking engine (e.g., AutoDock Vina-PyRx 0.8). A grid box was set around the ATP-binding site of HER2 to include the important catalytic residues [43]. Docking parameters were optimized to obtain an accurate sampling of the conformation of ligands. Binding affinity (kcal/mol) scores were recorded, and top-ranked poses were selected based on binding energy and the interaction profiles. Protein–ligand interactions were examined, which included hydrogen bonds, hydrophobic contacts, pi-pi, and electrostatic interactions. Compounds with good affinity for binding and good interaction patterns with critical active site residues were selected for further pharmacokinetic and dynamic studies [44]. The validation of the docking protocol was achieved by docking the native co-crystallized ligand within the HER2 active site [13].

### 4.7. ADME, Drug-like Property and Toxicity Predictions

The pharmacokinetic properties of the top-ranked compounds obtained by docking were assessed by in silico ADME prediction tools such as SwissADME [45]. Key parameters that were assessed were as follows:

- Adherence to Lipinski’s Rule of Five.
- Molecular weight.
- LogP (lipophilicity).
- Donors and acceptors of the hydrogen bond.
- Topological polar surface area (TPSA).
- The absorption into the gastrointestinal tract.
- Permeability of the blood–brain barrier.
- Profile of cytochrome P450 inhibition [46].

Drug likeness and toxicity risk parameters were also investigated to weed out compounds with unfavorable pharmacokinetic or safety properties. Compounds with acceptable ADME properties were selected for MD simulations to test the binding stability conditions under physiological conditions [47]. The toxicity prediction analysis was performed through ProTox 3.0.

### 4.8. pKa Prediction Analysis

The acid dissociation constant (pKa) values of the compounds selected were estimated using the MolGpKa web server, which is a graph neural network (GNN)-based predictor for pKa values of small molecules. The SDF files of the chemical structures of the compounds were created, and the SMILES were canonicalized before analysis. The compounds were submitted to MolGpKa, one by one, with the default prediction parameters. All ionizable functional groups were automatically identified, and their acidic and basic pKa values were predicted using graph-based molecular representation and deep learning algorithms trained on experimentally validated data. The pKa values of the compounds were then used to predict their predominant protonation states in physiological conditions (pH 7.4) to aid in the interpretation of molecular docking, MD simulations, and protein–ligand interaction analyses. Predictions were done with the default settings of the MolGpKa server without any modifications to the model [48].

### 4.9. Molecular Dynamics (MD) Simulation

Structural stability, conformational dynamics, and binding behavior of the HER2–ligand complexes have been assessed by all-atom molecular dynamics (MD) simulations for 200 ns for each complex with the AMBER24 simulation package. The protein was parameterized using the ff19SB force field, while the parameters for the ligands were generated using the General AMBER Force Field (GAFF2) through Antechamber. The partial atomic charges of the ligands were assigned during the parameterization of the ligand [49].

The protein–ligand complexes were solvated in an explicit OPC water box, with a minimum distance of 10 Å between the protein surface and the box boundaries. The system was neutralized with the addition of sodium ions to give an ionic strength of 0.15 M and minimized by the steepest descent algorithm to eliminate unfavorable steric contacts before equilibration [50].

The minimized systems were then slowly increased to a temperature of 300 K while pressure was kept constant (NVT), before equilibrating the systems at constant pressure (NPT) [47]. The temperature was controlled by the Langevin thermostat and the pressure by the Monte Carlo barostat (1 atm) [51]. Long-range electrostatic interactions were calculated with the Particle Mesh Ewald (PME) method, and short-range non-bonded interactions were calculated using a cutoff distance of 10 Å. After equilibration, 200 ns production runs were performed under periodic boundary conditions, and all the trajectories obtained were analyzed for structure and energy, such as RMSD, RMSF, radius of gyration, solvent-accessible surface area, hydrogen-bond analysis, principal component analysis, and MM/PBSA binding free-energy calculations [51].

### 4.10. Post-Simulation Analyses

Post-simulation analyses were performed and involved computation of root-mean-squared deviation (RMSD) to assess overall stability of the protein backbone and ligand, root-mean-squared fluctuation (RMSF) to assess the flexibility of residues, Beta Factor, and radius of gyration (Rog) to track the compactness of the protein [21]. Principal component analysis (PCA), dynamic cross-correlation matrix (DCCM), radial distribution function (RDF), secondary structure (SS), and salt bridges were performed as well. Hydrogen-bond interactions between HER2 and the ligands were analyzed to establish the number and duration of contacts, and binding free energy was estimated using the MM-PBSA approach to establish the thermodynamic favorability of ligand binding [52].

### 4.11. MM-PBSA Calculations

The estimated binding free energies of the protein–ligand complexes were obtained using the AMBER22 module called ‘MMPBSA.py’, which is based on the molecular mechanics/Poisson–Boltzmann surface area (MM/PBSA) method. The binding free energies were computed using equilibrated MD trajectories by taking snapshots at regular intervals to represent the trajectory [53]. The total binding free energy (ΔG_bind) was calculated as the sum of van der Waals, electrostatic, polar solvation, and non-polar solvation energy components. The internal and solvent dielectric constants were set to 1.0 and 80.0, respectively, for the MM/PBSA calculations. To obtain statistically reliable binding free-energy estimates, a total of 1000 snapshots were extracted from the production trajectory [22].

## 5. Conclusions

Altogether, the current study introduces a powerful computational pipeline to enhance the identification of promising HER2 inhibitors with the combination of ML, docking, and MD simulations. The agreement of our findings with the literature of computational studies is a strong confirmation that the methodology is sound and a good basis to support this by an experimental study. The next steps in future research will be improving the ML models, increasing MD simulation times, and performing empirical research on how the information obtained through these computational tools can be converted into effective therapeutic agents against HER2-positive breast cancer. We have used a combined computational approach that merged ML-based prioritization of compounds, molecular docking, and MD simulations to discover and screen potential breast cancer therapeutic HER2 inhibitors. The ML methodology was effective in screening a plethora of chemical libraries, which allowed us to focus on the best candidates. Follow-up docking experiments verified strong binding behavior of the three most promising compounds in the top selection with selected essential residues in the HER2 kinase domain, and MD analyses confirmed the stability and dynamic behavior of these interactions in similar physiological conditions. The three CHEMBL193865 (Lead-1), CHEMBL46740 (Lead-2), and CHEMBL151318 (Lead-3) chosen all exhibited favorable binding affinities, stable interactions, and positive conformational flexibility, which were better than the control compound in docking and MD analysis. Lead-2 was the compound that had the most favorable computational findings profile as compared to the other two leads. However, the results obtained have been interpreted with due precautions as they are based exclusively on computational analyses. Thus, Lead-2 should be considered a promising computationally found lead candidate, and not a validated HER2 inhibitor. It needs further in vitro and in vivo investigation to confirm its biological activity, pharmacological properties, and therapeutic potential.

The consecutive combination of ML, docking, and MD thus provides a potent platform for rational drug discovery, improved predictive accuracy, and lowered computational resources. In general, the article has revealed three potential future HER2-targeting compounds, which can be further tested in experiments, including cell-based and in vitro kinase assays. The model research achieved in this study can be used to inform the rapid development of targeted therapeutics in HER2-positive breast cancer and other oncogenic targets to close the gap between computational prediction and experimental development.

## Figures and Tables

**Table 1:** Contains the compound details, including the chemical structures/names, along with the binding affinities.

S. No	Compounds IDs	Chemical Structure	Chemical Name	Binding Affinity
1	CHEMBL193865 (Lead-1)	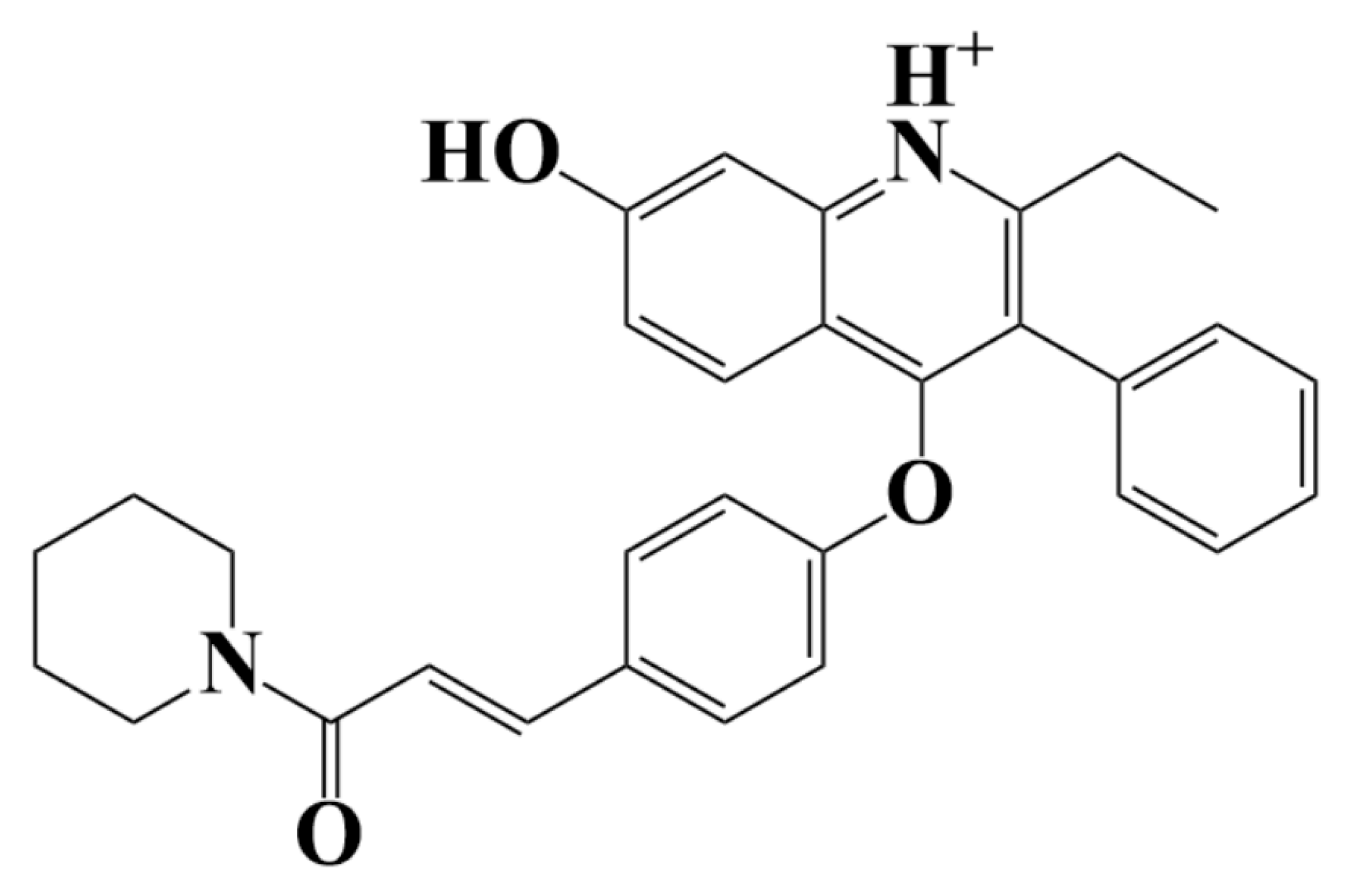	(E)-2-ethyl-7-hydroxy-4-(4-(3-oxo-3-(piperidin-1-yl)prop-1-en-1-yl)phenoxy)-3-phenylquinolin-1-ium	−12.1 kcal/mol
2	CHEMBL46740 (Lead-2)	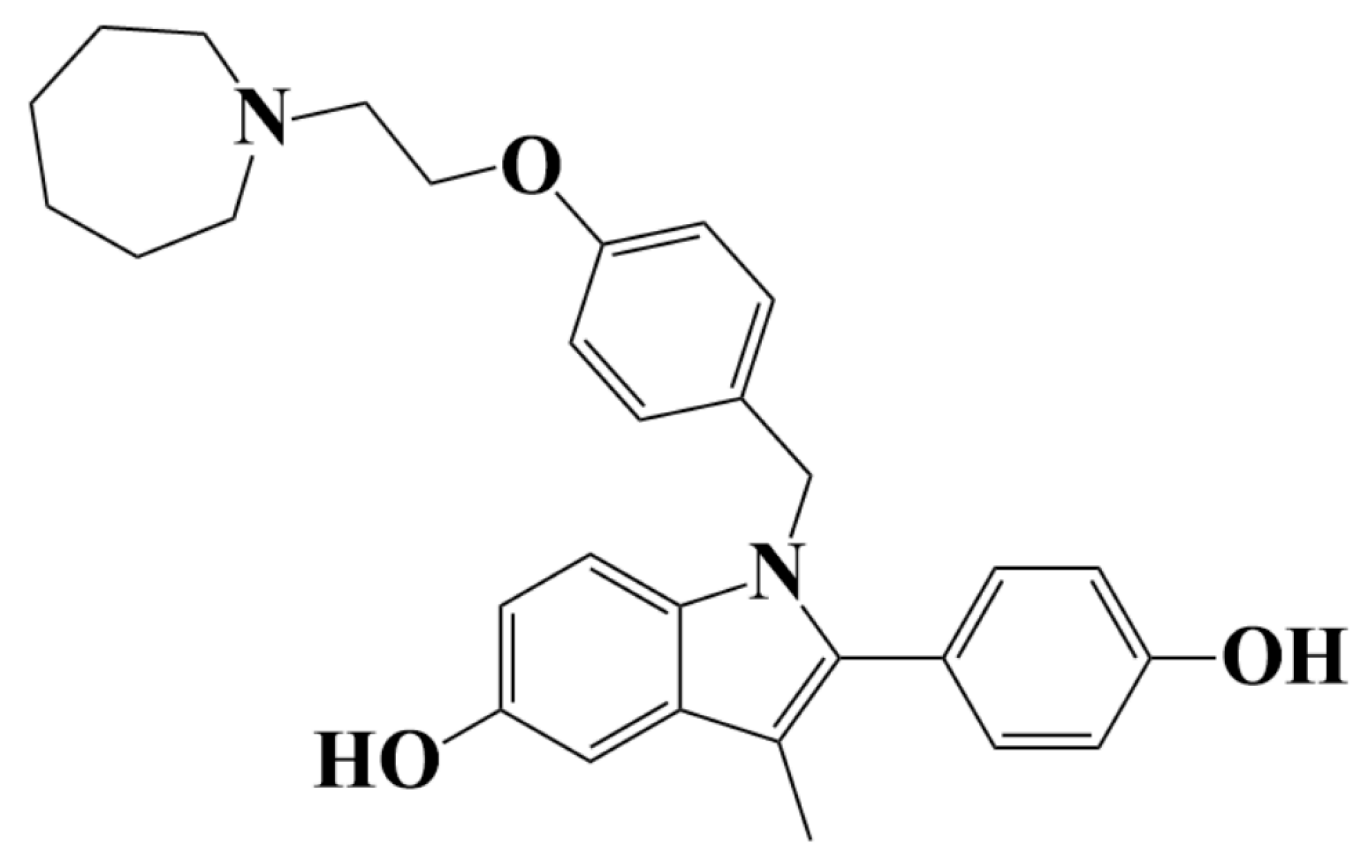	11-(4-(2-(azepan-1-yl)ethoxy)benzyl)-2-(4-hydroxyphenyl)-3-methyl-1H-indol-5-ol	−11.8 kcal/mol
3	CHEMBL151318 (Lead-3)	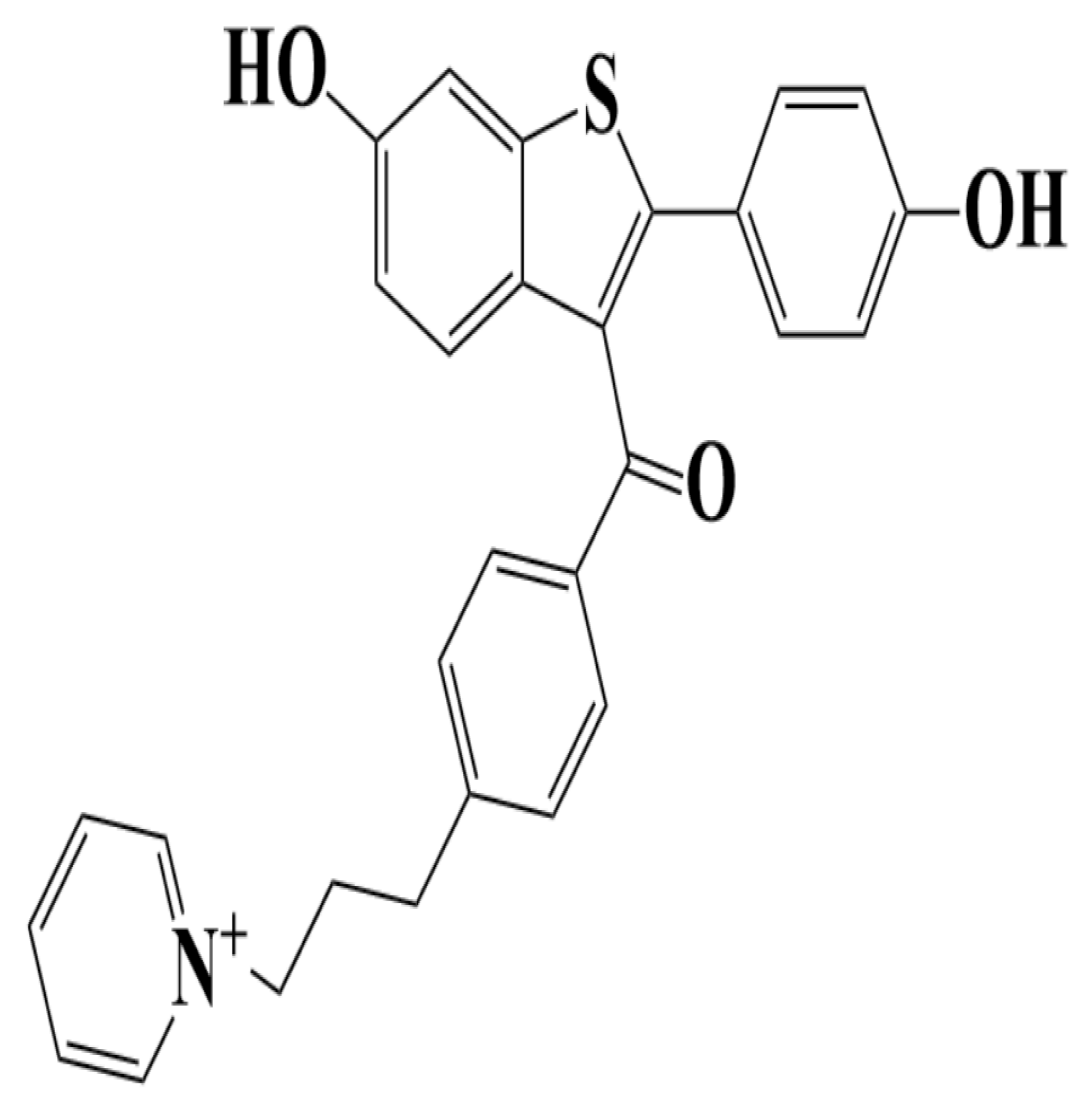	1-(3-(4-(6-hydroxy-2-(4-hydroxyphenyl)benzo[b]thiophene-3-carbonyl)phenyl)propyl)pyridin-1-ium	−11.7 kcal/mol
4	Control	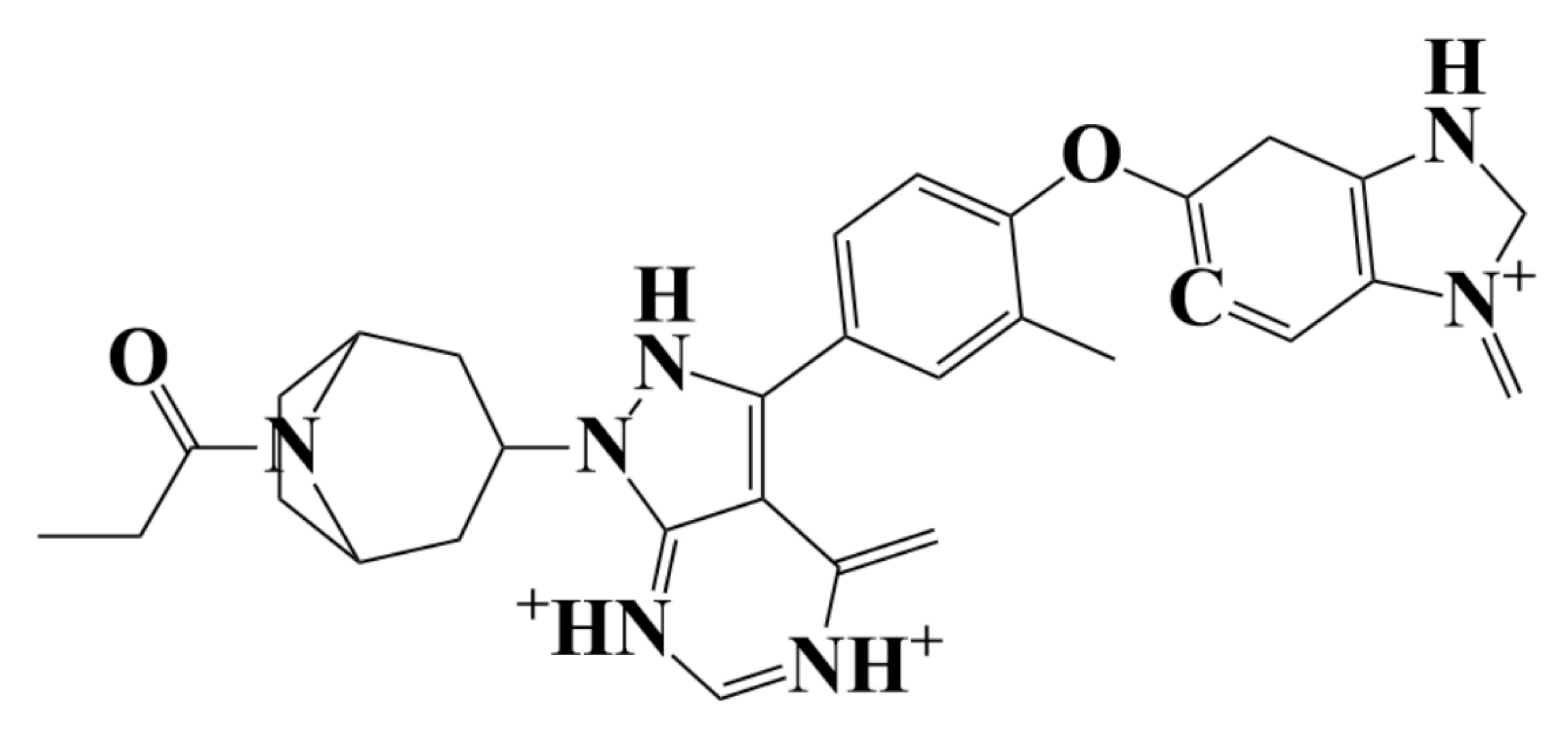	5-(2-methyl-4-(4-methylene-1-(8-propionyl-8-azabicyclo[3.2.1]octan-3-yl)-2,4-dihydro-1H-indazol-3-yl)phenoxy)-1-methylene-2,3,4,5,6,7-hexahydro-1H-benzo[d]imidazol-1-ium	−10.8 kcal/mol

**Table 2:** Provides the pKa values of the compounds and control.

Compound	Major Ionizable Group(s)	Acidic pKa (Phenolic OH)	Basic pKa (Amine/Nitrogen)	Predicted Net Charge at pH 7.4	Major Species at pH 7.4
Control	Phenolic Oh, Amidine/Imidazoline-like N	9.5–10.0	7.2–8.0	0 To +1	Neutral/Partially Protonated
Lead-1	Phenolic Oh, Heterocyclic Nh	9.2–10.2	4.5–6.0	0	Predominantly Neutral
Lead-2	Two Phenolic Oh Groups, Piperidine Tertiary Amine	9.3–10.2	10.5–11.2	+1	Monocation
Lead-3	Two Phenolic Oh Groups, Piperidine Tertiary Amine	9.2–10.1	10.6–11.2	+1	Monocation

**Table 3:** Offers details on Hydrogen bonds involving the residues of the HER2 kinase domain and the three different ligands of interest: CHEMBL193865 (Lead-1), CHEMBL46740 (Lead-2), CHEMBL151318 (Lead-3), and the control ligand (D).

Lead-1						
#Acceptor	DonorH	Donor	Frames	Frac	AvgDist	AvgAng
ASP_99@OD2	LIG_309@H	LIG_309@O2	103	0.1035	2.7738	154.7306
ASP_99@OD1	LIG_309@H	LIG_309@O2	79	0.0794	2.7614	157.2898
SER_17@OG	LIG_309@HN	LIG_309@N	3	0.003	2.9107	156.7691
ARG_140@O	LIG_309@H	LIG_309@O2	1	0.001	2.9796	163.2434
**Lead-2**						
#Acceptor	DonorH	Donor	Frames	Frac	AvgDist	AvgAng
SER_74@O	LIG_309@H1	LIG_309@O2	609	0.6121	2.7234	158.6114
SER_74@OG	LIG_309@H1	LIG_309@O2	78	0.0784	2.8632	156.7848
**Lead-3**						
#Acceptor	DonorH	Donor	Frames	Frac	AvgDist	AvgAng
MET_92@O	LIG_309@H	LIG_309@O1	163	0.1638	2.8159	148.8382
LIG_309@O1	MET_92@H	MET_92@N	9	0.009	2.9441	144.2772
ASN_141@OD1	LIG_309@H1	LIG_309@O2	5	0.005	2.8551	148.468
GLN_90@O	LIG_309@H	LIG_309@O1	3	0.003	2.8751	143.2886
SER_17@O	LIG_309@H1	LIG_309@O2	1	0.001	2.7241	137.9225
**Control**						
#Acceptor	DonorH	Donor	Frames	Frac	AvgDist	AvgAng
TYR_94@O	LIG_309@H3	LIG_309@N5	4	0.004	2.8834	153.5994
ARG_102@NH1	LIG_309@HN	LIG_309@N	1	0.001	2.9498	138.0479

**Table 4:** The binding free energy (MMPBSA) of the three compounds CHEMBL193865 (A), CHEMBL46740 (B), CHEMBL151318 (C), and control (D).

Technique	Energy Section	Lead-1	Lead-2	Lead-3	Control
MMPBSA	Van der Waals Energy (kcal/mol)	−105.36	−98.23	−95.66	−90.73
	Electrostatic Energy (kcal/mol)	−25.30	−24.00	−27.65	−20.19
	Polar Salvation Energy (SE) (kcal/mol)	28.42	30.69	35.09	30.19
	Non-Polar SE (kcal/mol)	−5.06	−6.30	−5.76	−5.33
	Gas Phase Energy (kcal/mol)	−130.66	−122.23	−123.31	−110.92
	**Total** (kcal/mol)	−107.3	−97.84	−93.98	−86.06

## Data Availability

The original contributions presented in this study are included in the article/Supplementary Materials. Further inquiries can be directed to the corresponding author.

## Supplementary Materials

The following supporting information can be downloaded at https://www.mdpi.com/article/10.3390/ph19081190/s1, Table S1: Performance metrics for four classification models (Random Forest, SVM, KNN, XGBoost) evaluated on the test set and a holdout set, including AUC, PR-AUC, accuracy, precision, recall, F1-score, and Matthews correlation coefficient (MCC). Figure S1. The ADME properties of Lead-1. Figure S2. The ADME properties of Lead-2. Figure S3. The ADME properties of Lead-3. Figure S4. The ADME properties of control. Figure S5. Toxicity detail of Lead-1 (A). Figure S6. Toxicity detail of Lead-2 (B). Figure S7. Toxicity detail of Lead-3 (C). Figure S8: pKa values and details of the three Leads and Control. Lead-1 (A), Lead-2 (B), Lead-3 (C) and Control (D). Figure S9 Representative conformational snapshots of the HER2–Lead-1 complex obtained at the beginning (0 ns) and the end (200 ns) of the molecular dynamics simulation. Figure S10. Representative conformational snapshots of the HER2–Lead-2 complex obtained at the beginning (0 ns) and the end (200 ns) of the molecular dynamics simulation. Figure S11. Representative conformational snapshots of the HER2–Lead-3 complex obtained at the beginning (0 ns) and the end (200 ns) of the molecular dynamics simulation. Figure S12. Representative conformational snapshots of the HER2–control complex obtained at the beginning (0 ns) and the end (200 ns) of the molecular dynamics simulation.
