# Supplementary material for: Machine Learning-Driven Discovery of Novel HER2 Inhibitors Through Integrated Virtual Screening and Molecular Dynamics Simulations
Source: Pharmaceuticals (Basel). 2026 Jul 29;19(8):1190. doi: 10.3390/ph19081190 (PMC13516331; doi:10.3390/ph19081190)
Supplement: Supplementary file 1 [file pharmaceuticals-19-01190-s001.zip › pharmaceuticals-4426317-supplementary.pdf]

Supplementary File

Table S1: Performance metrics for four classification models (Random Forest, SVM, KNN, XGBoost) evaluated on the test set and a holdout set, including AUC, PR-AUC, accuracy, precision, recall, F1-score, and Matthews correlation coefficient (MCC)

Model	Test AUC	Test PR-AUC	Test Accuracy	Test Precision	Test Recall	Test F1	Test MCC	Holdout AUC	Holdout PR-AUC	Holdout Accuracy	Holdout Precision	Holdout Recall	Holdout F1	Holdout MCC
Random Forest	0.95	0.91	0.94	0.93	0.9	0.91	0.86	0.82	0.75	0.8	0.78	0.72	0.75	0.62
SVM	0.84	0.77	0.85	0.81	0.78	0.79	0.68	0.82	0.74	0.79	0.75	0.71	0.73	0.59
KNN	0.83	0.75	0.83	0.77	0.76	0.76	0.65	0.82	0.73	0.78	0.73	0.7	0.71	0.56
XGBoost	0.72	0.62	0.78	0.71	0.65	0.67	0.52	0.73	0.61	0.76	0.68	0.62	0.65	0.48

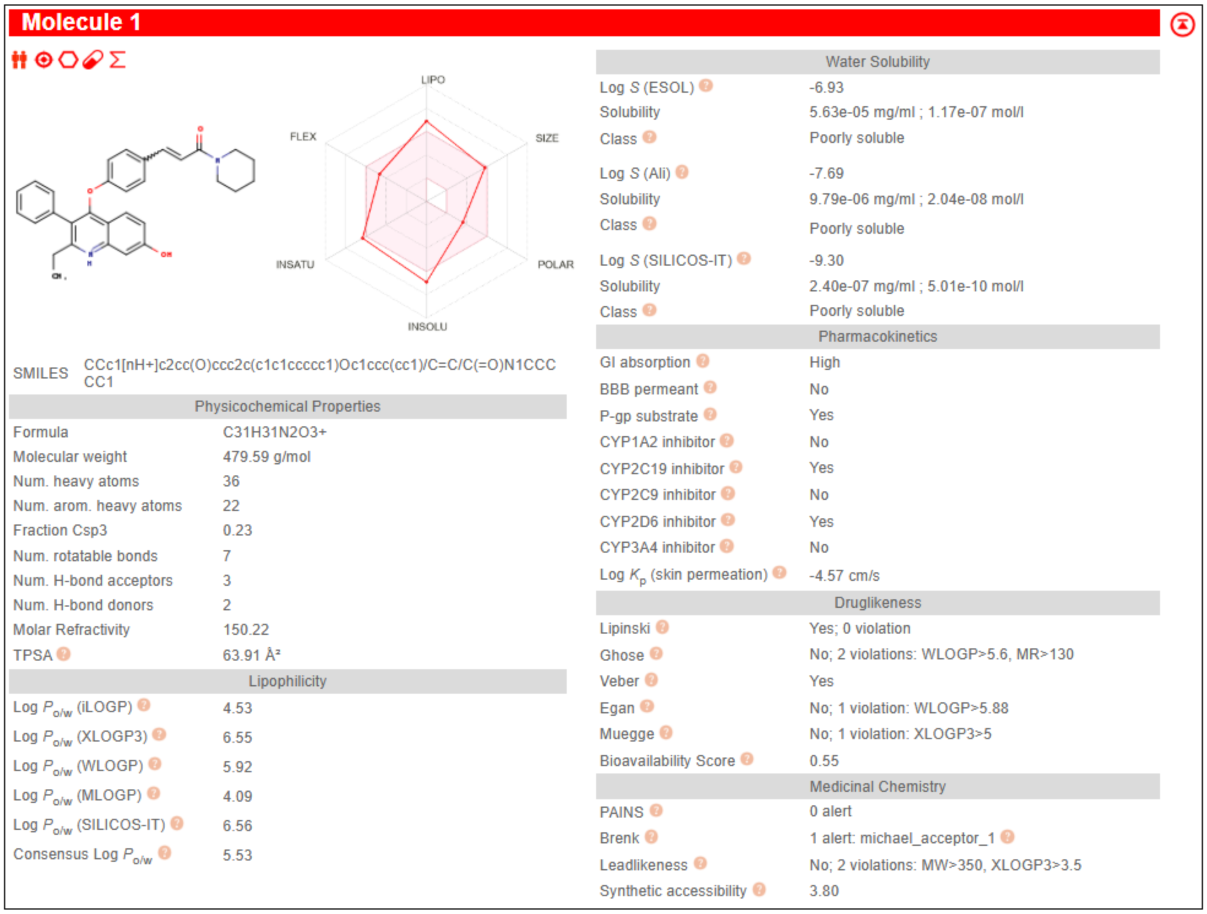

Figure S1. The ADME properties of Lead-1

Molecule 1

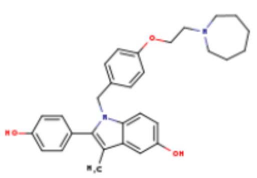

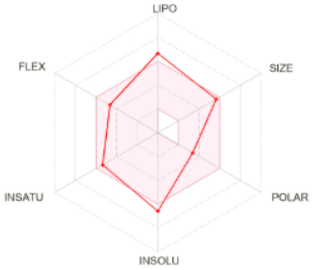

SMILES Oc1ccc2c(c1)c(C)c(n2Cc1ccc(cc1)OCCN1CCCCCC1)c1ccc(cc1)O

Physicochemical Properties	
Formula	C30H34N2O3
Molecular weight	470.60 g/mol
Num. heavy atoms	35
Num. arom. heavy atoms	21
Fraction Csp3	0.33
Num. rotatable bonds	7
Num. H-bond acceptors	4
Num. H-bond donors	2
Molar Refractivity	146.97
TPSA	57.86 Å²

Lipophilicity	
Log $P_{o/w}$ (iLOGP)	4.06
Log $P_{o/w}$ (XLOGP3)	6.12
Log $P_{o/w}$ (WLOGP)	5.95
Log $P_{o/w}$ (MLOGP)	3.67
Log $P_{o/w}$ (SILICOS-IT)	5.58
Consensus Log $P_{o/w}$	5.07

Water Solubility	
Log *S* (ESOL)	-6.60
Solubility	1.19e-04 mg/ml ; 2.54e-07 mol/l
Class	Poorly soluble
Log *S* (Ali)	-7.12
Solubility	3.59e-05 mg/ml ; 7.64e-08 mol/l
Class	Poorly soluble
Log *S* (SILICOS-IT)	-8.54
Solubility	1.35e-06 mg/ml ; 2.88e-09 mol/l
Class	Poorly soluble

Pharmacokinetics	
GI absorption	High
BBB permeant	No
P-gp substrate	Yes
CYP1A2 inhibitor	No
CYP2C19 inhibitor	Yes
CYP2C9 inhibitor	No
CYP2D6 inhibitor	Yes
CYP3A4 inhibitor	No
Log K_p (skin permeation)	-4.83 cm/s

Druglikeness	
Lipinski	Yes; 0 violation
Ghose	No; 2 violations: WLOGP>5.6, MR>130
Veber	Yes
Egan	No; 1 violation: WLOGP>5.88
Muegge	No; 1 violation: XLOGP3>5
Bioavailability Score	0.55

Medicinal Chemistry	
PAINS	0 alert
Brenk	0 alert
Leadlikeness	No; 2 violations: MW>350, XLOGP3>3.5
Synthetic accessibility	3.48

Figure S2. The ADME properties of Lead-2

Molecule 1

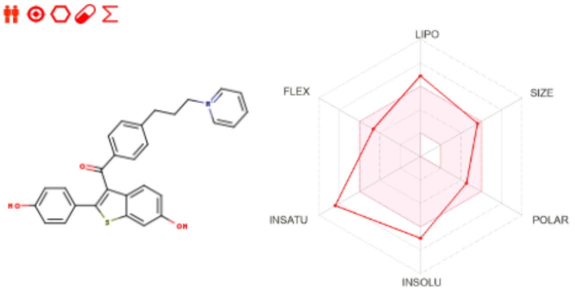

SMILES Oc1ccc2c(c1)sc(c2C(=O)c1ccc(cc1)CCC[n+]1ccccc1)c1ccc(cc1)O

Physicochemical Properties	
Formula	C29H24NO3S+
Molecular weight	466.57 g/mol
Num. heavy atoms	34
Num. arom. heavy atoms	27
Fraction Csp3	0.10
Num. rotatable bonds	7
Num. H-bond acceptors	3
Num. H-bond donors	2
Molar Refractivity	138.87
TPSA	89.65 Å²

Lipophilicity	
Log $P_{o/w}$ (iLOGP)	-0.17
Log $P_{o/w}$ (XLOGP3)	6.54
Log $P_{o/w}$ (WLOGP)	6.13
Log $P_{o/w}$ (MLOGP)	3.76
Log $P_{o/w}$ (SILICOS-IT)	7.01
Consensus Log $P_{o/w}$	4.65

Water Solubility	
Log *S* (ESOL)	-6.98
Solubility	4.90e-05 mg/ml ; 1.05e-07 mol/l
Class	Poorly soluble
Log *S* (Ali)	-8.22
Solubility	2.81e-06 mg/ml ; 6.02e-09 mol/l
Class	Poorly soluble
Log *S* (SILICOS-IT)	-9.57
Solubility	1.26e-07 mg/ml ; 2.69e-10 mol/l
Class	Poorly soluble

Pharmacokinetics	
GI absorption	High
BBB permeant	No
P-gp substrate	Yes
CYP1A2 inhibitor	Yes
CYP2C19 inhibitor	Yes
CYP2C9 inhibitor	No
CYP2D6 inhibitor	No
CYP3A4 inhibitor	Yes
Log K_p (skin permeation)	-4.50 cm/s

Druglikeness	
Lipinski	Yes; 0 violation
Ghose	No; 2 violations: WLOGP>5.6, MR>130
Veber	Yes
Egan	No; 1 violation: WLOGP>5.88
Muegge	No; 1 violation: XLOGP3>5
Bioavailability Score	0.55

Medicinal Chemistry	
PAINS	0 alert
Brenk	1 alert: quaternary_nitrogen_1
Leadlikeness	No; 2 violations: MW>350, XLOGP3>3.5
Synthetic accessibility	3.52

Figure S3. The ADME properties of Lead-3

Molecule 1

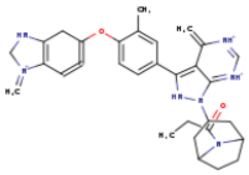

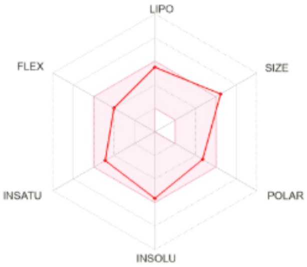

SMILES CCC(=O)N1C2CCC1CC(C2)n1[nH]c(c2c1[nH+]c[nH+]c2=C)c1ccc(c(c1)C)OC1=C=CC2=C(C1)NC[N+]2=C

Physicochemical Properties	
Formula	C31H36N7O2+++
Molecular weight	538.66 g/mol
Num. heavy atoms	40
Num. arom. heavy atoms	15
Fraction Csp3	0.39
Num. rotatable bonds	6
Num. H-bond acceptors	2
Num. H-bond donors	4
Molar Refractivity	169.39
TPSA	93.58 Å²

Lipophilicity	
Log $P_{o/w}$ (iLOGP)	0.00
Log $P_{o/w}$ (XLOGP3)	4.12
Log $P_{o/w}$ (WLOGP)	1.53
Log $P_{o/w}$ (MLOGP)	3.04
Log $P_{o/w}$ (SILICOS-IT)	3.80
Consensus Log $P_{o/w}$	2.50

Water Solubility	
Log *S* (ESOL)	-5.66
Solubility	1.19e-03 mg/ml ; 2.20e-06 mol/l
Class	Moderately soluble
Log *S* (Ali)	-5.79
Solubility	8.70e-04 mg/ml ; 1.62e-06 mol/l
Class	Moderately soluble
Log *S* (SILICOS-IT)	-7.64
Solubility	1.22e-05 mg/ml ; 2.27e-08 mol/l
Class	Poorly soluble

Pharmacokinetics	
GI absorption	High
BBB permeant	No
P-gp substrate	Yes
CYP1A2 inhibitor	No
CYP2C19 inhibitor	No
CYP2C9 inhibitor	No
CYP2D6 inhibitor	No
CYP3A4 inhibitor	Yes
Log K_p (skin permeation)	-6.66 cm/s

Druglikeness	
Lipinski	Yes; 1 violation: MW>500
Ghose	No; 3 violations: MW>480, MR>130, #atoms>70
Veber	Yes
Egan	Yes
Muegge	Yes
Bioavailability Score	0.55

Medicinal Chemistry	
PAINS	0 alert
Brenk	1 alert: quaternary_nitrogen_1
Leadlikeness	No; 2 violations: MW>350, XLOGP3>3.5
Synthetic accessibility	6.59

Figure S4. The ADME properties of Control

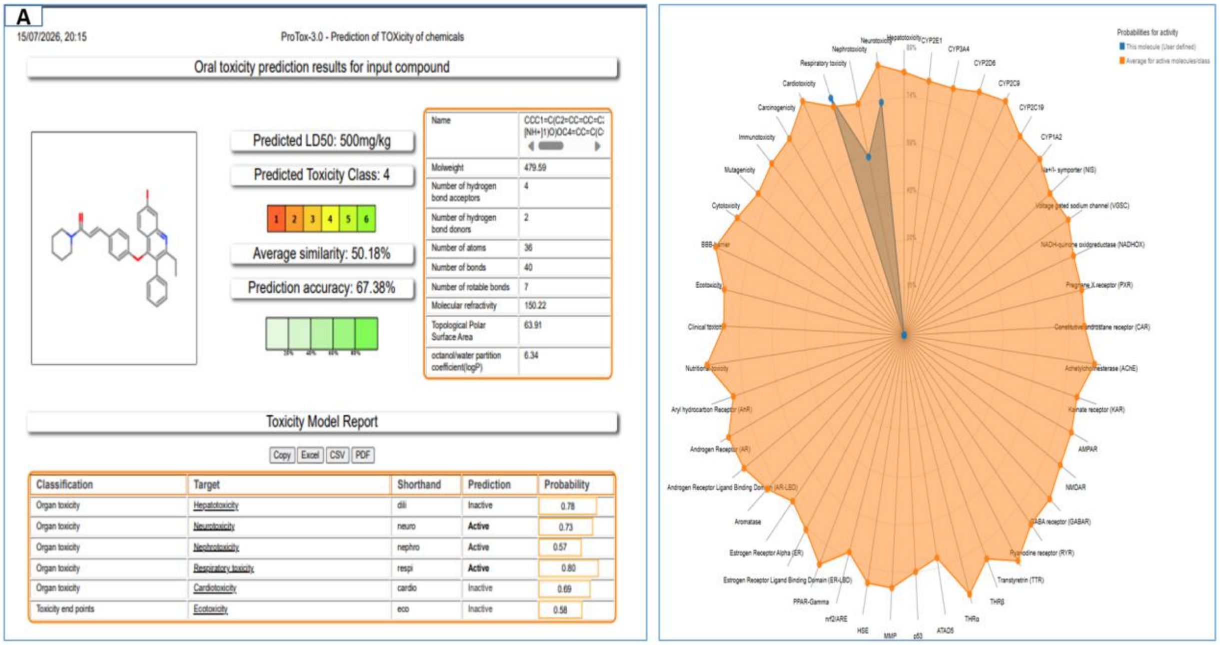

Figure S5. Toxicity detail of Lead-1 (A)

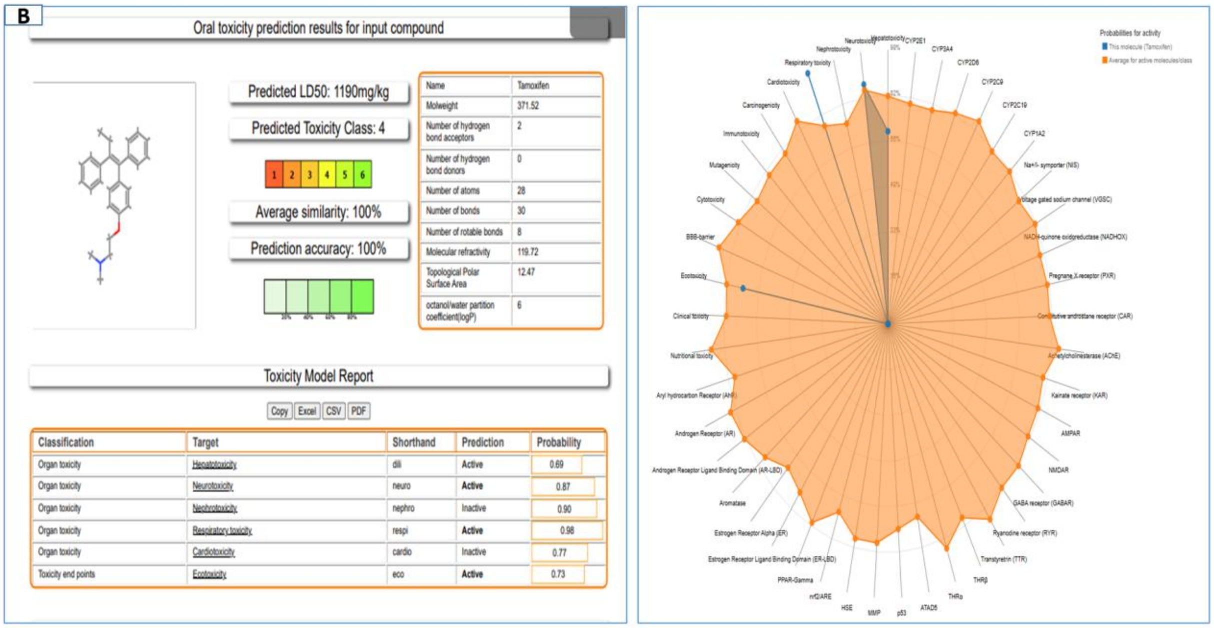

Figure S6. Toxicity detail of Lead-2 (B)

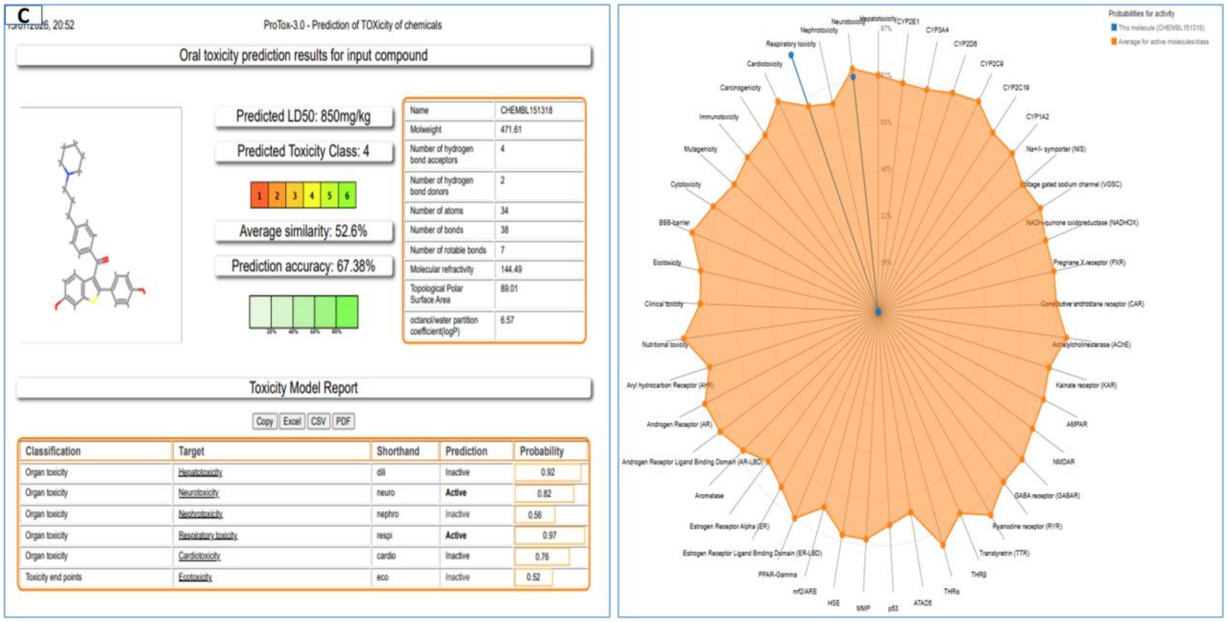

Figure S7. Toxicity detail of Lead-3 (C)

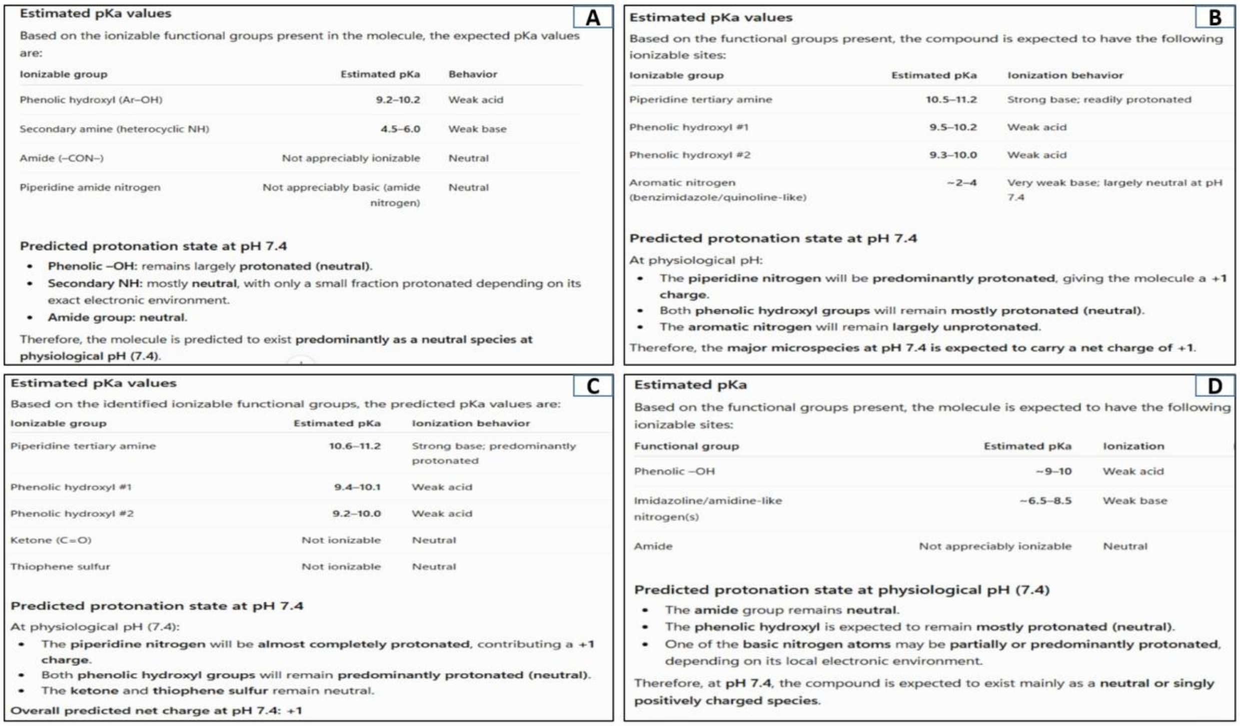

A

Estimated pKa values

Based on the ionizable functional groups present in the molecule, the expected pKa values are:

Ionizable group	Estimated pKa	Behavior
Phenolic hydroxyl (Ar–OH)	9.2–10.2	Weak acid
Secondary amine (heterocyclic NH)	4.5–6.0	Weak base
Amide (–CON–)	Not appreciably ionizable	Neutral
Piperidine amide nitrogen	Not appreciably basic (amide nitrogen)	Neutral

Predicted protonation state at pH 7.4

- **Phenolic –OH:** remains largely **protonated (neutral)**.
- **Secondary NH:** mostly **neutral**, with only a small fraction protonated depending on its exact electronic environment.
- **Amide group: neutral.**

Therefore, the molecule is predicted to exist **predominantly as a neutral species at physiological pH (7.4).**

B

Estimated pKa values

Based on the functional groups present, the compound is expected to have the following ionizable sites:

Ionizable group	Estimated pKa	Ionization behavior
Piperidine tertiary amine	10.5–11.2	Strong base; readily protonated
Phenolic hydroxyl #1	9.5–10.2	Weak acid
Phenolic hydroxyl #2	9.3–10.0	Weak acid
Aromatic nitrogen (benzimidazole/quinoline-like)	~2–4	Very weak base; largely neutral at pH 7.4

Predicted protonation state at pH 7.4

At physiological pH:

- The **piperidine nitrogen** will be **predominantly protonated**, giving the molecule a **+1 charge**.
- Both **phenolic hydroxyl groups** will remain **mostly protonated (neutral)**.
- The **aromatic nitrogen** will remain **largely unprotonated**.

Therefore, the **major microspecies at pH 7.4 is expected to carry a net charge of +1**.

C

Estimated pKa values

Based on the identified ionizable functional groups, the predicted pKa values are:

Ionizable group	Estimated pKa	Ionization behavior
Piperidine tertiary amine	10.6–11.2	Strong base; predominantly protonated
Phenolic hydroxyl #1	9.4–10.1	Weak acid
Phenolic hydroxyl #2	9.2–10.0	Weak acid
Ketone (C=O)	Not ionizable	Neutral
Thiophene sulfur	Not ionizable	Neutral

Predicted protonation state at pH 7.4

At physiological pH (7.4):

- The **piperidine nitrogen** will be **almost completely protonated**, contributing a **+1 charge**.
- Both **phenolic hydroxyl groups** will remain **predominantly protonated (neutral)**.
- The **ketone** and **thiophene sulfur** remain neutral.

Overall predicted net charge at pH 7.4: +1

D

Estimated pKa

Based on the functional groups present, the molecule is expected to have the following ionizable sites:

Functional group	Estimated pKa	Ionization
Phenolic –OH	~9–10	Weak acid
Imidazoline/amidine-like nitrogen(s)	~6.5–8.5	Weak base
Amide	Not appreciably ionizable	Neutral

Predicted protonation state at physiological pH (7.4)

- The **amide** group remains **neutral**.
- The **phenolic hydroxyl** is expected to remain **mostly protonated (neutral)**.
- One of the **basic nitrogen atoms** may be **partially or predominantly protonated**, depending on its local electronic environment.

Therefore, at **pH 7.4**, the compound is expected to exist mainly as a **neutral or singly positively charged species.**

Figure S8: pKa values and details of the three Leads and Control. Lead-1 (A), Lead-2 (B), Lead-3 (C) and Control (D).

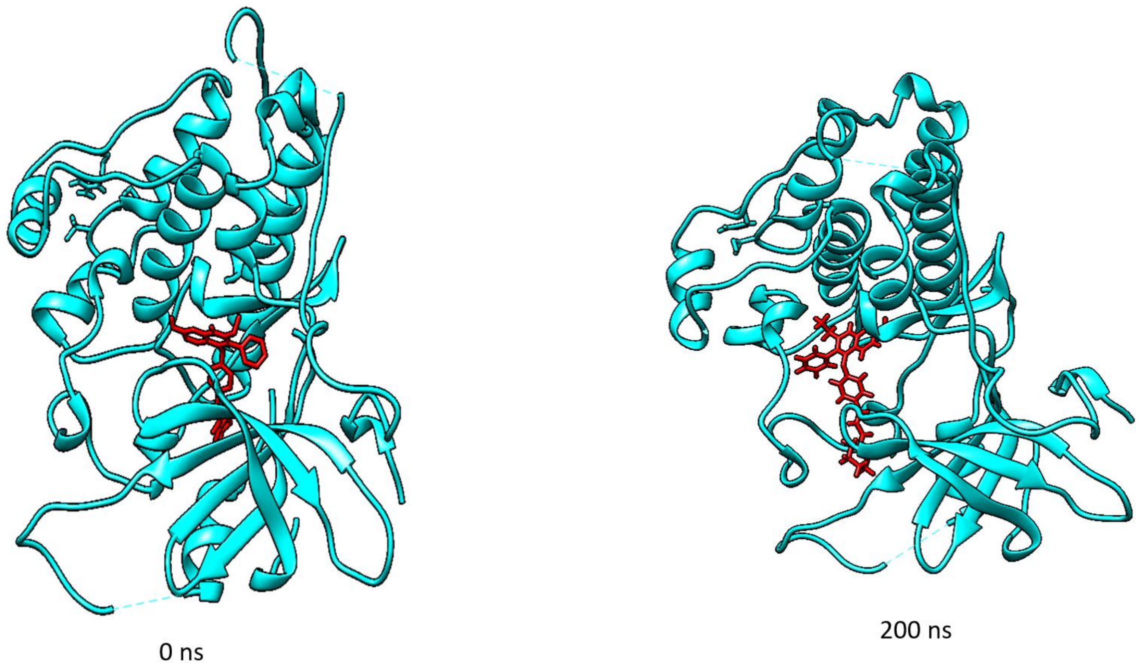

Figure S9 Representative conformational snapshots of the HER2–Lead-1 complex obtained at the beginning (0 ns) and the end (200 ns) of the molecular dynamics simulation

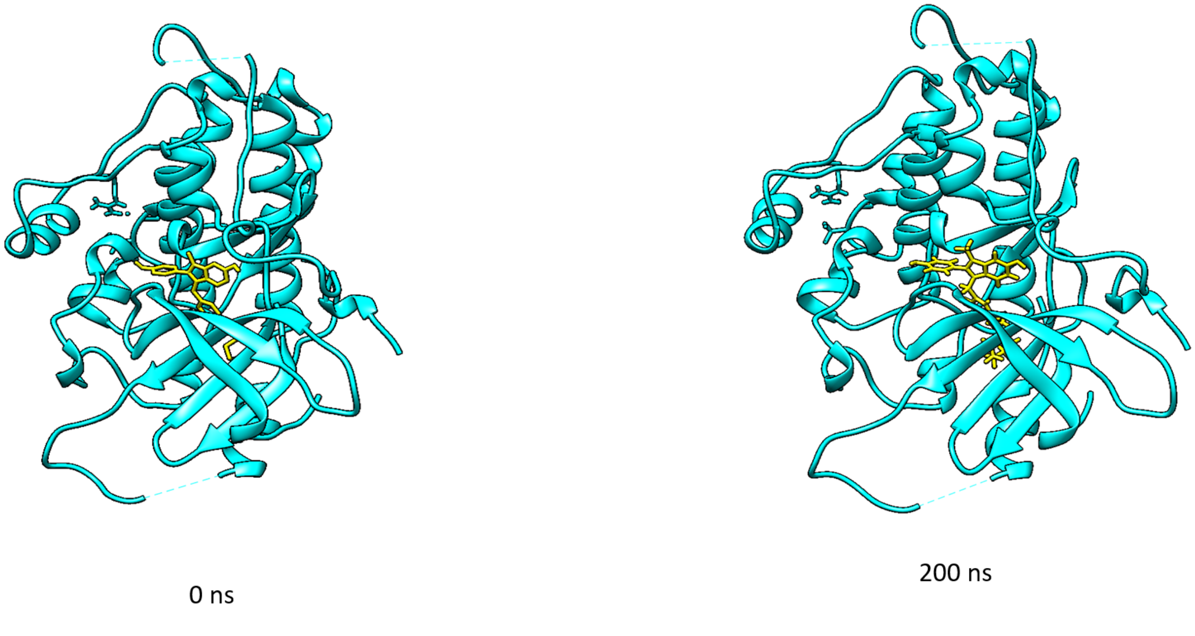

Figure S10. Representative conformational snapshots of the HER2–Lead-2 complex obtained at the beginning (0 ns) and the end (200 ns) of the molecular dynamics simulation.

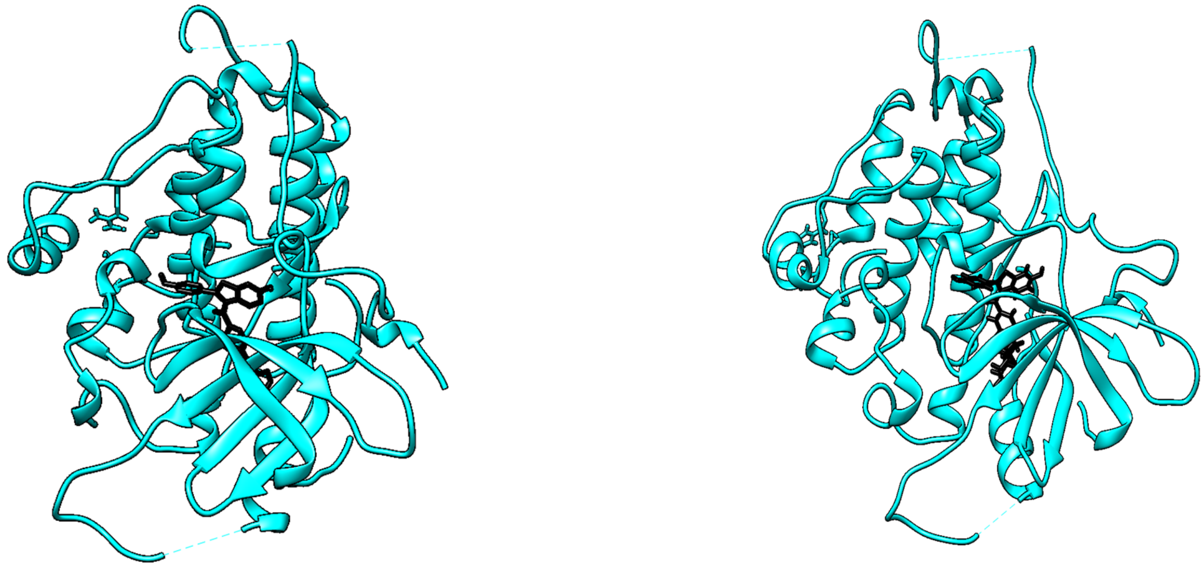

0 ns

200 ns

Figure S11. Representative conformational snapshots of the HER2–Lead-3 complex obtained at the beginning (0 ns) and the end (200 ns) of the molecular dynamics simulation

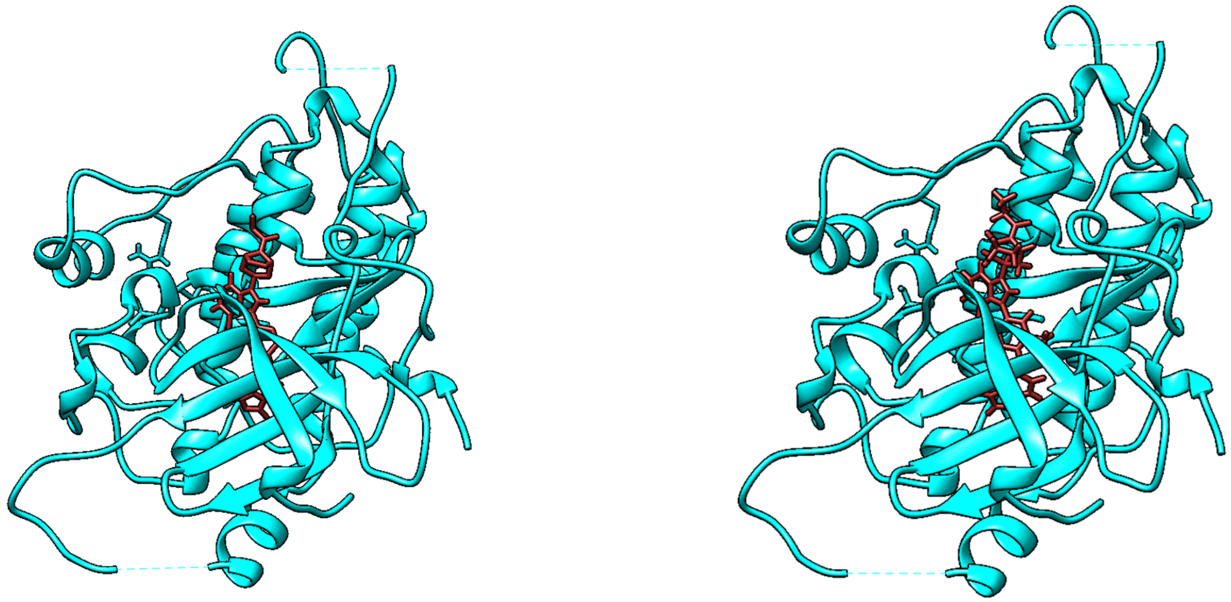

0 ns

200 ns

Figure S12. Representative conformational snapshots of the HER2–control complex obtained at the beginning (0 ns) and the end (200 ns) of the molecular dynamics simulation.
